# Beta-Carotene Reduces Body Adiposity of Mice via BCMO1

**DOI:** 10.1371/journal.pone.0020644

**Published:** 2011-06-01

**Authors:** Jaume Amengual, Erwan Gouranton, Yvonne G. J. van Helden, Susanne Hessel, Joan Ribot, Evelien Kramer, Beata Kiec-Wilk, Ursula Razny, Georg Lietz, Adrian Wyss, Aldona Dembinska-Kiec, Andreu Palou, Jaap Keijer, Jean François Landrier, M. Luisa Bonet, Johannes von Lintig

**Affiliations:** 1 Laboratory of Molecular Biology, Nutrition and Biotechnology, Universitat de les Illes Balears and CIBER de Fisiopatología de la Obesidad y Nutrición (CIBERobn), Palma de Mallorca, Spain; 2 Department of Pharmacology, School of Medicine, Case Western Reserve University, Cleveland, Ohio United States of America; 3 INRA, UMR 1260 Nutriments Lipidiques et Prevention des Maladies Métaboliques/Universite Aix-Marseille I et II, Marseille, France; 4 Human and Animal Physiology, Wageningen University, Wageningen, The Netherlands; 5 University Maastricht, Maastricht, The Netherlands; 6 RIKILT-Institute of Food Safety, Wageningen, The Netherlands; 7 Institute of Biology I, University of Freiburg, Freiburg, Germany; 8 Department of Clinical Biochemistry, The Jagiellonian University Medical College, Krakow, Poland; 9 School of AFRD, Newcastle University, Newcastle upon Tyne, United Kingdom; 10 DSM Nutritional Products, R&D Human Nutrition and Health, Kaiseraugst, Switzerland; The University of Kansas Medical center, United States of America

## Abstract

Evidence from cell culture studies indicates that β-carotene-(BC)-derived apocarotenoid signaling molecules can modulate the activities of nuclear receptors that regulate many aspects of adipocyte physiology. Two BC metabolizing enzymes, the BC-15,15′-oxygenase (Bcmo1) and the BC-9′,10′-oxygenase (Bcdo2) are expressed in adipocytes. Bcmo1 catalyzes the conversion of BC into retinaldehyde and Bcdo2 into β-10′-apocarotenal and β-ionone. Here we analyzed the impact of BC on body adiposity of mice. To genetically dissect the roles of Bcmo1 and Bcdo2 in this process, we used wild-type and *Bcmo1* ^-/-^ mice for this study. In wild-type mice, BC was converted into retinoids. In contrast, *Bcmo1^-/-^* mice showed increased expression of *Bcdo2* in adipocytes and β-10′-apocarotenol accumulated as the major BC derivative. In wild-type mice, BC significantly reduced body adiposity (by 28%), leptinemia and adipocyte size. Genome wide microarray analysis of inguinal white adipose tissue revealed a generalized decrease of mRNA expression of peroxisome proliferator-activated receptor γ (PPARγ) target genes. Consistently, the expression of this key transcription factor for lipogenesis was significantly reduced both on the mRNA and protein levels. Despite β-10′-apocarotenoid production, this effect of BC was absent in *Bcmo1^-/-^* mice, demonstrating that it was dependent on the Bcmo1-mediated production of retinoids. Our study evidences an important role of BC for the control of body adiposity in mice and identifies Bcmo1 as critical molecular player for the regulation of PPARγ activity in adipocytes

## Introduction

In mammals, β-carotene (BC) is the natural precursor for apocarotenoids molecules including retinoids (vitamin A and its derivatives) [1]. Two different types of BC metabolizing enzymes have been identified that are expressed in various tissues [2]-[4]. The β-carotene-15,15′-monooxygenase (Bcmo1) converts BC to all-*trans*-retinal [5]; [6] and studies in knockout (*Bcmo1* ^-/-^) mice show that Bcmo1 is the primary enzyme for retinoid production [7]-[9]. Besides Bcmo1, mammalian genomes encode a second BC metabolizing enzyme known as β-carotene-9′,10′-dioxygenase (Bcdo2). Bcdo2 cleaves BC at position 9,10, resulting in the formation of one molecule β-ionone and one molecule β-10′-apo-carotenal [4].

Increasing evidence has been provided that BC-derived apocarotenoid signaling molecule can influence adipocyte physiology. *Bcmo1* knockout mice are highly susceptible to high fat diet-induced obesity and show increased expression of peroxisome proliferator-activated receptor γ (PPARγ) regulated genes in fat depots [7]. PPARs control the expression of genes for lipid and glucose metabolism [10]; [11] and PPARγ is pivotal for adipocyte differentiation and lipogenesis in mature adipocytes [12]. *Bcmo1* gene expression is under the control of PPARγ [13]; [14] and is induced during adipocyte differentiation [15]. The primary BC cleavage product retinaldehyde has been shown to inhibit PPARγ activity both in adipocyte cell cultures and mouse models [16]. Furthermore, evidence has been provided that Bcmo1 plays an important role for retinoic acid production and retinoic acid receptor (RAR) signaling in adipocytes [15]. Retinoic acid influences adipocyte differentiation [17]; [18] and fat deposition [19], mitochondrial uncoupling [20]; [21], oxidative metabolism [22]; [23] and the expression of adipokines such as leptin, resistin, and serum retinol binding protein [24]-[27]. Part of these effects are mediated via RARs, which upon retinoic acid binding regulate the expression of direct target genes [28] and interfere with the activity of other transcription factors, including early adipogenic transcription factors such as PPARγ [17]; [18]. In addition, retinoic acid may influence PPAR-mediated responses by activating the retinoid X receptor (RXR) moiety of permissive PPAR:RXR heterodimers [29] and, possibly, by serving as an agonist of PPARβ/δ [30]; [31]. Finally, BC-derived long chain apocarotenoids such as β-apo-14′-carotenal can inhibit PPARγ activity and adipogenesis in cell culture [32]. Moreover, β-13-apocarotenone has been shown to inhibit RXRα activity [33].

These findings implicate that a tissue-specific conversion of BC via carotenoid-oxygenases can influence the activities of key transcription factor that control adipocyte physiology. However, this concept lacks experimental testing in animal models. Here we wished to systematically analyze the effects of BC on the adipose phenotype in mice. To genetically dissect the contributions of Bcmo1 and Bcdo2 to this process, we used wild-type (WT) and *Bcmo1* ^-/-^ mice in this study. Our analysis included a genome wide expression analysis of inguinal white adipose tissue (iWAT) to identify the target genes of BC action in this tissue.

## Results

### Effects of dietary BC supplementation on BC and retinoid levels in serum and adipose tissue

**Figure 1:**
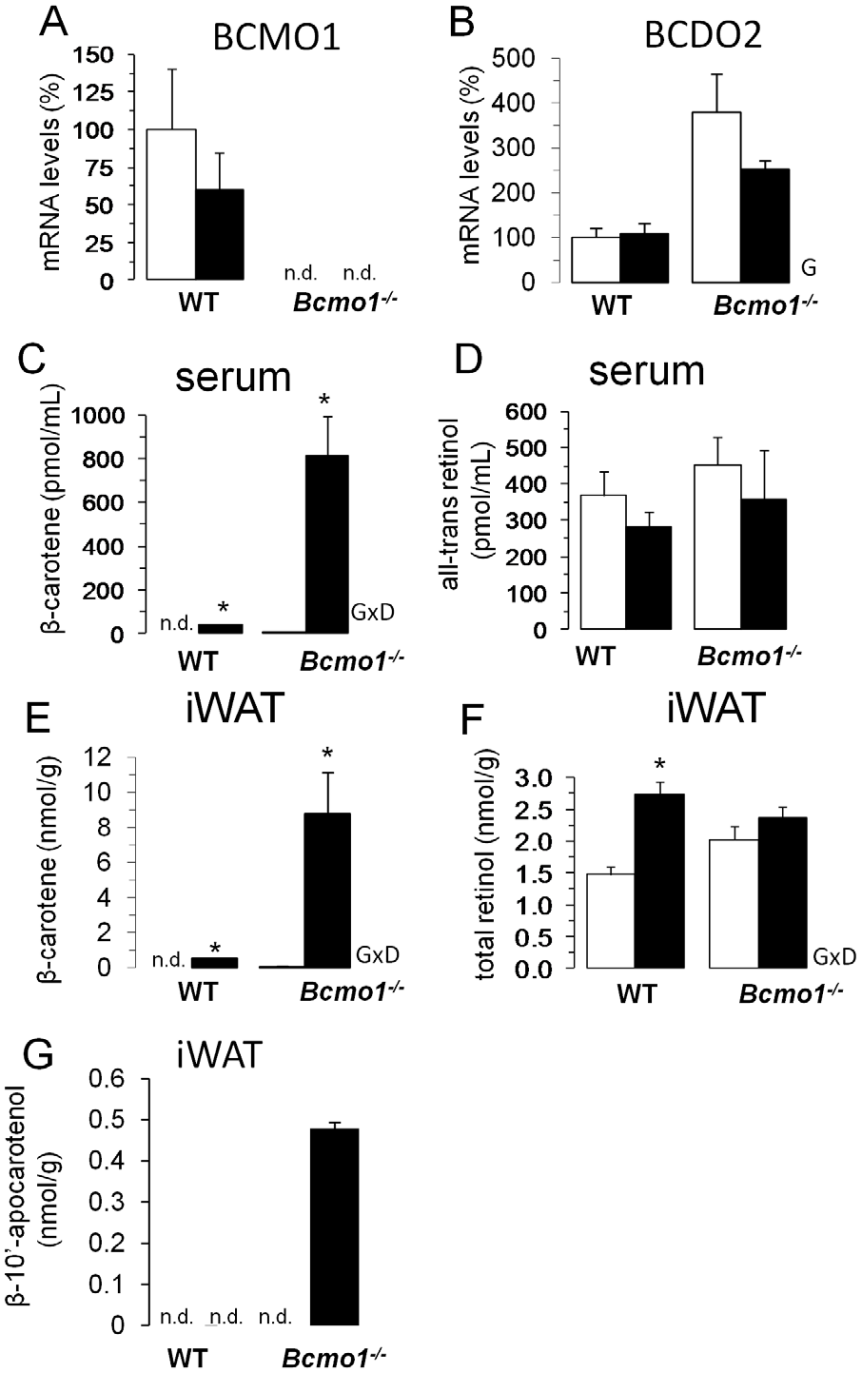
Serum and inguinal white adipose tissue levels of β-carotene, retinoids and apocarotenoids. (**A**, **B**) β-carotene -15, 15′-monoxygenase (Bcmo1) and β-carotene 9′, 10′-dioxygenase (Bcdo2) mRNA levels in inguinal white adipose tissue (iWAT) of wild-type (WT) and *Bcmo1*-null (*Bcmo1* ^-/-^) mice after 14 weeks on a control diet (open bars) or a β-carotene-enriched diet (black bars). Quantitative real-time PCR was used to determine normalized gene expression levels. (**C**-**G**) β-carotene, retinol and 10′ β-apocarotenol levels in serum and iWAT of WT and *Bcmo1* ^-/-^ mice after 14 weeks on a control diet (open bars) or a β-carotene-enriched diet (black bars). β-carotene, retinol and β-10′-apocarotenol were determined by HPLC (see Materials and Methods). Total retinol refers to free retinol plus retinyl esters. Data in (**A** to **G**) are the mean ± SEM of 6 animals per group; n.d. non-detectable; GxD, interaction between genotype and diet in two-way ANOVA analysis (p<0.05); G, genotype effect in two-way ANOVA analysis (p<0.05); *, p<0.05 in Student's *t* test, BC diet *versus* control diet.

Five-week-old WT and *Bcmo1* ^-/-^ female mice were fed a defined, pelletized diet containing 1500 IU vitamin A/kg with (BC diet) or without (control diet) 150 mg BC/kg diet (n = 6 animals per group). After 14 weeks, we first studied expression of *Bcmo1* in iWAT of different dietary groups and genotypes. *Bcmo1* was expressed in iWAT of WT but not *Bcmo1* ^-/-^ mice (Figure 1A). We also analyzed the mRNA expression levels of *Bcdo2,* which encodes a second carotenoid metabolizing enzyme [5]. This analysis revealed a marked up-regulation of *Bcdo2* expression in iWAT of *Bcmo1*-null mice, both with control and BC diet (Figure 1B). Similar results were obtained for *Bcmo1* and *Bcdo2* expression in gonadal WAT (Figure S1).

**Figure 2:**
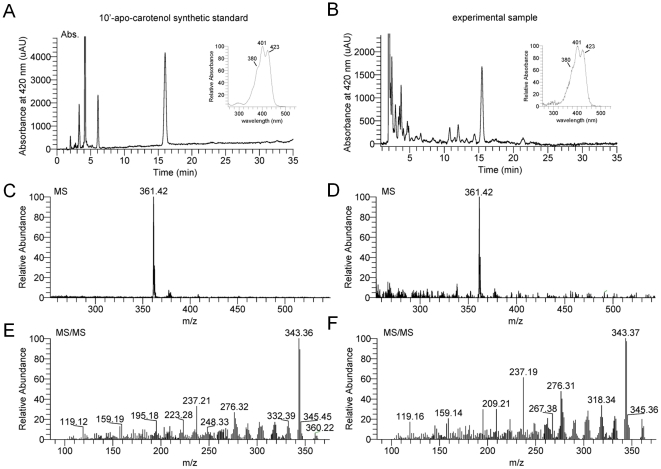
β-10′-apocarotenol is the major β-carotene cleavage product in *Bcmo1^-/-^* mice. HPLC-profile at 420 nm of (**A**) the synthetic β-10′-apocarotenol standard and (**B**) a lipid extract of iWAT of BC supplemented *Bcmo1^-/-^* mice. Insets show the spectral characteristics of the β-10′-apocarotenol standard (peak 1) as compared to peak 1′. (**C**, **D**) Single extracted ion chromatograms for β-10′-apocarotenol standard (m/z  = 361.42; peak 1 and 1′). (**E**, **F**) Fragmentation pattern of a parent ion selected at m/z  = 361.42 are identical for the β-10′-apocarotenol standard (peak 1) and peak 1′.

Serum and iWAT BC levels were significantly increased after the 14 weeks of dietary intervention in all animals subjected to BC supplementation (Figures 1C and 1E). This increase was much more pronounced in the *Bcmo1* ^-/-^ mice, in which BC accumulation was readily evidenced by an orange coloring of the fat depots (not shown). Serum retinol levels were similar regardless of genotype or diet (Figure 1D). iWAT total retinol levels (free retinol plus retinyl ester) were increased upon the BC diet in WT but not in *Bcmo1* ^-/-^ mice (Figure 1F). In *Bcmo1^-/-^* mice supplemented with BC, a compound that migrated shortly after all-*trans*-retinol with an absorption maximum of 402 nm was present (Fig. 1G). Spectral characteristics and retention time indicated that this compound is β-10′-apocarotenol, the corresponding alcohol of the primary Bcdo2 cleavage product β-10′-apo-carotenal. To confirm this hypothesis, we used a β-10′-apocarotenal standard and reduced it to the corresponding alcohol by BH_4_ treatment. In fact, this β-10′-apocarotenol standard had the same retention time and spectral characteristics of the compound that accumulated in *Bcmo1^-/-^* mice upon BC supplementation (Fig 2A and 2B). Finally, we confirmed the identity of this compound by tandem mass spectrometry as compared β-10′-apocarotenol standard substance (Fig. 2C-F). Thus, we concluded that in WT mice supplemented BC is largely converted to retinoids. When BC accumulated in *Bcmo1^-/-^* mice, it is converted in part to β-10′-apocarotenal that is further reduced to the corresponding alcohol.

### Dietary BC reduces adiposity in WT mice

**Figure 3:**
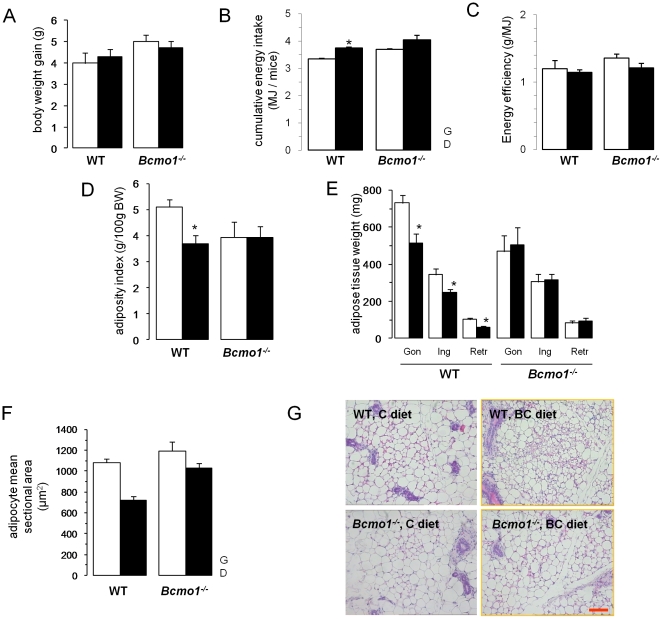
Supplemetation of β-carotene alters body adiposity in wild-type mice. (**A**) Effects of dietary β-carotene supplementation on body weight gain, (**B**) cumulative energy intake, (**C**) energy efficiency, (**D**) adiposity index, (**E**) adipose depot weight, and (**F**) inguinal adipocyte mean sectional area, in wild-type (WT) and *Bcmo1*-null (*Bcmo1* ^-/-^) mice. In (**G**), micrographs of sections of inguinal fat in the four groups are shown (The scale bar gives 100 µm). Data in (**A** to **E**) are the mean±SEM of 6 animals per group, and data in (**F**) of 3 animals per group Open bars, animals on the control diet; black bars, animals on the β-carotene-enriched diet. Body weight gain, cumulative energy intake and energy efficiency over the entire 14 week experimental period are shown. Adiposity index corresponds to the sum of all entirely dissected white adipose tissue depots (gonadal, Gon; inguinal, Ing; and retroperitoneal, Retr), expressed as percentage of the animal body weight (BW).. G, genotype effect in two-way ANOVA analysis (p<0.05); D, effect of diet in two-way ANOVA analysis (p<0.05);*, p<0.05 in Student's *t* test, BC diet versus control diet.

*Bcmo1* ^-/-^ mice had a lower average body weight than WT mice at the beginning of the study (17.3±0.4 g versus 18.5±0.2 g; p<0.05 Student's t test) and showed a tendency of higher body weight gain over the 14 week experimental period (Figure 3A). Body weight at the end of the study was comparable in all groups (data not shown). Cumulative energy intake over the 14 week experimental period was significantly higher in mice fed the BC diet than in those fed the control diet, and in *Bcmo1* ^-/-^ mice as compared to WT mice (Figure 3B). Energy efficiency (body weight gained per energy eaten) was similar among groups (Figure 3C). Even though BC supplementation did not affect final body weight in either genotype, it significantly decreased the percentage of body fat (by 28%, p<0.01) in WT but not in *Bcmo1* ^-/-^ mice (Figure 3D). In WT mice, reduction of depot mass after BC diet was evident for all three WAT depots examined (inguinal, gonadal and retroperitoneal) (Figure 3E); no such effect of BC on fat depots was observed in the *Bcmo1* ^-/-^ mice. Leptin levels in serum reflected changes in body adiposity, being reduced following dietary BC supplementation in the WT mice but not in the *Bcmo1* ^-/-^ mice (Table 1). There was a direct, positive correlation between serum leptin levels and iWAT mass (r = 0.617, p<0.001). Morphometric analysis showed that the mean sectional area of unilocular iWAT adipocytes was reduced after dietary BC supplementation in the WT mice (Figure 3F and 3G). Mean iWAT adipocyte area was similar in the two genotype groups after the control diet, but significantly higher in the *Bcmo1* ^-/-^ mice after the BC diet. Circulating levels of insulin, glucose and triacylglycerol were unaffected by diet or genotype (Table 1).

**Table 1:** Fed blood parameters in wild-type and *Bcmo1^-/-^* mice following control and β-carotene enriched diet.

	WT control diet	WT BC diet	*Bcmo1^-/-^* control diet	*Bcmo1^-/-^* BC diet	Anova
Glucose (mg/dL)	211±22	220±9	242±12	248±21	
Triacylglycerol (mg/mL)	0.31±0.05	0.30±0.03	0.31±0.05	0.40±0.08	
Insulin (ng/mL)	0.47±0.20	0.36±0.10	0.49±0.15	0.59±0.18	
Leptin (ng/mL)	9.63±1.69	4.09±0.59*	6.72±1.74	7.33±1.59	GxD

Analysis were carried out in wild-type (WT) and *Bcmo1^-/-^* female mice fed for 14 weeks a control diet or a β-carotene-enriched diet (BC diet), starting at 5 weeks of age. Data are the mean ± SEM of 6 animals per group. *, p<0.05 in Student's t test, BC diet versus control diet; GxD, interaction between genotype and diet in two-way ANOVA analysis (p<0.05).

### Dietary BC results in a general down-regulation of gene expression in adipose tissue of WT mice

**Figure 4:**
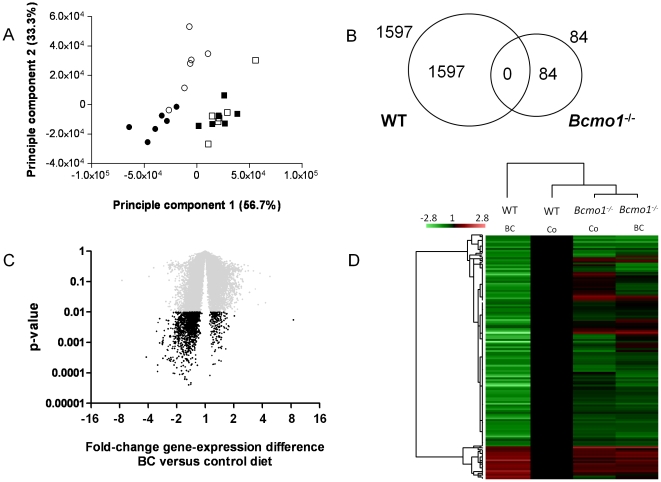
Supplementation of β-carotene alters gene expression profiles in wild-type but not in *Bcmo1^-/-^* mice. (**A**) Principal component analysis of transcriptional profiles in inguinal white adipose tissue of female wild-type (circles) and *Bcmo1* ^-/-^ (squares) mice fed the control diet (open symbols) or the β-carotene diet (closed symbols). Genome-wide expression profiles were obtained using the 4×44 k Agilent whole mouse genome microarrays for each animal in the experiment (n = 6 per group). (**B**) Venn diagram representing the number of significantly regulated genes (p<0.01) due to BC supplementation in wild-type (WT) and in *Bcmo1*-null (*Bcmo1* ^-/-^) mice and the number of significantly regulated genes regardless of genotype (overlap). (**C**) Volcano plot representing the effect of BC supplementation on gene expression in WT mice, with the fold-change on the x-axis and the corresponding Student's t-test p-value on the y-axis. Every spot is a single gene and in black are all genes with a p-value <0.01. (**D**) Heat map representing the expression level of all genes regulated by BC with a Student's t-test p<0.01 in the WT mice in all four groups; WT control (Co) diet, WT BC diet, *Bcmo1* ^-/-^ control diet and *Bcmo1* ^-/-^ BC diet. Relative expression of every single gene was compared to gene expression in WT mice on the control diet and consequently, gene expression of every single gene in WT mice on the control diet was set to 1.0.

To analyze the effects of BC supplementation on transcriptional activities in adipose tissue in different genotypes we performed genome-wide expression analysis of iWAT of all individual animals. Principal component analysis of microarray results revealed a different gene expression signature in WT and *Bcmo1*-null mice, and a clear effect of dietary BC supplementation that changed the overall gene expression signature in the WT mice, but not in the *Bcmo1*-null mice (Figure 4A). From the 25139 spots with signal intensities above two times the background, 1597 positive spots were differentially expressed due to BC supplementation in the WT mice and only 84 in the *Bcmo1*-null mice (p<0.01) (Figure 4B). These 84 positive spots correspond to 81 genes, that are given in Table S1 and are likely regulated by BC or β-10′-apocarotenoid accumulation. None of the genes affected by BC supplementation in the WT mice (p<0.01) were affected in the *Bcmo1*-null mice (Figure 4B, 0 genes overlapping). In the WT mice, genes significantly regulated (p<0.01) by BC supplementation appeared to differ most in expression as compared to the other three groups; WT control diet, *Bcmo1* ^-/-^ control diet and *Bcmo1* ^-/-^ BC diet (Figure 4D). Remarkably, upon BC supplementation, 87% of the genes affected in WT mice were found to be down-regulated (Figures 4C and 4D). Pathway analysis using Metacore showed that genes encoding components of lipid and glucose metabolism-related pathways (including cholesterol biosynthesis, fatty acid metabolism, glycerophospholipid metabolism, pentose phosphate pathway, pyruvate metabolism and glycolysis/ gluconeogenesis) as well as ATP synthesis and oxidative phosphorylation were among the most affected (Table S2). Because of the close connections between neovascularisation and adipose tissue expansion [34]–[35], we specifically examined changes in the expression of angiogenesis-related genes. As expected, BC supplementation triggered a generalized down-regulation of these genes in the WT mice, but not in the *Bcmo1* ^-/-^ mice (Table S3).

### Dietary BC down-regulates PPARγ and PPARγ target genes in adipose tissue of WT mice

**Figure 5:**
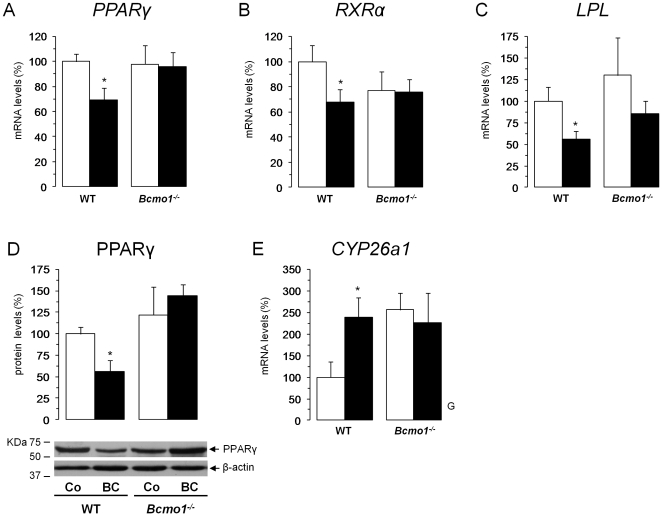
β-carotene supplementation reduces PPARγ expression both on the mRNA and protein level. (**A**) *PPARγ* mRNA levels, (**B**) *RXRα* mRNA levels, (**C**) *lipoprotein lipase* (*LPL*) mRNA levels, (**D**) PPARγ protein levels and (**E**) *Cyp26a1* mRNA levels in inguinal white adipose tissue of wild-type (WT) and *Bcmo1*-null (*Bcmo1* ^-/-^) mice after 14 weeks on a control diet (open bars) or a β-carotene -enriched diet (black bars). Quantitative real-time PCR was used to determine normalized gene expression levels as described in Materials and Methods. Immunoblotting was used to determine expression levels of PPARγ and β-actin, which was used as internal control for equal loading and blotting; shown at the bottom of (**D**) is a representative immunoblot for the two proteins. Data in (**A** to **E**) are the mean ± SEM of 6 animals per group. G, effect of genotype in two-way ANOVA analysis (p<0.05); *, p<0.05 in Student's t test, BC diet versus control diet.

A general effect of BC supplementation in reducing global transcriptional activity in iWAT of WT mice suggested that the activity of a master control transcription factor of adipose tissue biology was affected. In this context, a batch promoter analysis with Eldorado coupled to matinspector (Genomatix Suite) revealed that 54% of the top 50 down-regulated genes in iWAT of BC supplemented WT mice harbored a putative PPAR response element in their promoter region (p value: 1.33e^−6^) with at least 85% homology with the matrix similarity defined by Genomatix (Table 2). Interestingly, the same kind of analysis in iWAT of BC supplemented *Bcmo1^-/-^* mice revealed that the presence of putative PPAR responsive element was not significant (p value: 0.81). Microarray results indicated that iWAT gene expression of PPARγ and its heterodimeric partner RXRα were significantly decreased following BC supplementation in the WT mice (–1.75 and −1.57 fold-change, respectively, p<0.05), but not in the *Bcmo1* ^-/-^ mice. Q-PCR analysis confirmed the microarray results and thus down-regulation of the mRNA expression levels of PPARγ and RXRα after BC supplementation in iWAT of WT mice, but not of *Bcmo1* ^-/-^ mice (Figure 5A and 5B). Additionally, PPARγ protein expression levels in iWAT followed the same pattern as the mRNA levels, as indicated by immunoblotting results (Figure 5D). In contrast, iWAT mRNA levels of CYP26a1, a retinoic acid hydroxylase that is transcriptionally induced by retinoic acid in a strictly RAR-dependent manner [36]; [37], were increased by 138% after BC supplementation in the WT mice, but not in the *Bcmo1* ^-/-^ mice (Figure 5E), suggesting a Bcmo1-dependent enhancement of retinoic acid signalling in iWAT following chronic BC supplementation. Finally, to provide direct evidence for down-regulation of PPARγ activity after BC supplementation in the WT mice, we determined iWAT mRNA levels of LPL, a well-known target of PPARγ:RXRα, by Q-RT-PCR. As shown in Fig. 5C, LPL mRNA levels were significantly decreased in WT mice upon BC supplementation. Changes in gene expression of PPARγ, RXRα and LPL in the gonadal fat depot followed the same pattern as in the inguinal depot (Figure S1).

**Table 2:** Batch promoter analysis focused on peroxisome proliferator-activated receptor (PPAR) of the fifty top down-regulated genes in inguinal white adipose tissue of wild-type mice after β-carotene supplementation.

Gene	Gene name	p value	fold-change	putative PPRE
Npr3	natriuretic peptide receptor 3	0.00033	−4.18	Yes
Mosc1	MOCO sulphurase C-terminal domain containing 1	0.00234	−3.26	Yes
Dnmt3l	DNA (cytosine-5-)-methyltransferase 3-like	0.00452	−3.14	
Scd2	stearoyl-Coenzyme A desaturase 2	0.00606	−3.02	Yes
Lep	Leptin	0.00106	−2.84	
Rbp4	retinol binding protein 4, plasma	0.00030	−2.80	Yes
Pnpla3	patatin-like phospholipase domain containing 3	0.00098	−2.78	
Dgat2	diacylglycerol O-acyltransferase 2	0.00166	−2.68	Yes
Slc2a5	solute carrier family 2 (facilitated glucose transporter), member 5	0.02198	−2.60	
Fads3	fatty acid desaturase 3	0.00027	−2.52	Yes
Alb1	albumin 1	0.00058	−2.51	Yes
Mod1	malic enzyme, supernatant	0.00280	−2.46	Yes
Gsta3	glutathione S-transferase, alpha 3	0.00075	−2.46	
Mogat2	monoacylglycerol O-acyltransferase 2	0.00408	−2.44	Yes
Fabp5	fatty acid binding protein 5, epidermal	0.00026	−2.37	
Elovl6	ELOVL family member 6, elongation of long chain fatty acids (yeast)	0.01515	−2.36	Yes
Ela1	elastase 1, pancreatic	0.00155	−2.35	Yes
Cav1	caveolin, caveolae protein 1	0.00083	−2.35	
Cspg3	chondroitin sulfate proteoglycan 3	0.02156	−2.33	
Apol6	apolipoprotein L, 6	0.00167	−2.33	Yes
LOC676546	similar to monocyte to macrophage differentiation-associated	0.00024	−2.32	Yes
Sncg	synuclein, gamma	0.00155	−2.32	
Ppp2r5b	protein phosphatase 2, regulatory subunit B (B56), beta isoform	0.00294	−2.31	
Tkt	Transketolase	0.01153	−2.31	
Ntsr2	neurotensin receptor 2	0.00032	−2.31	Yes
Acss2	acyl-CoA synthetase short-chain family member 2	0.01181	−2.28	Yes
Mid1ip1	Mid1 interacting protein 1 (gastrulation specific G12-like (zebrafish))	0.03037	−2.21	Yes
Retn	Resistin	0.00282	−2.19	
Lpgat1	lysophosphatidylglycerol acyltransferase 1	0.00024	−2.19	Yes
Lctl	lactase-like	0.00010	−2.18	Yes
Slc6a13	solute carrier family 6 (neurotransmitter transporter, GABA), member 13	0.00394	−2.17	
Comt	catechol-O-methyltransferase	0.00946	−2.17	
Thbd	Thrombomodulin	0.01020	−2.17	Yes
Orm2	orosomucoid 2	0.00026	−2.14	Yes
Orm1	orosomucoid 1	0.00018	−2.13	Yes
Lrrc39	leucine rich repeat containing 39	0.00130	−2.12	
Sc5d	sterol-C5-desaturase (fungal ERG3, delta-5-desaturase) homolog (S. cerevisae)	0.00095	−2.12	
Fabp4	fatty acid binding protein 4, adipocyte	0.00690	−2.11	Yes
Lss	lanosterol synthase	0.00103	−2.10	
Odz4	odd Oz/ten-m homolog 4 (Drosophila)	0.00063	−2.10	
Slc9a2	solute carrier family 9 (sodium/hydrogen exchanger), member 2	0.00214	−2.10	Yes
Aldh1a7	aldehyde dehydrogenase family 1, subfamily A7	0.00010	−2.10	Yes
Cpa2	carboxypeptidase A2, pancreatic	0.00157	−2.10	
Timp4	tissue inhibitor of metalloproteinase 4	0.01792	−2.09	
Slc2a4	solute carrier family 2 (facilitated glucose transporter), member 4	0.01228	−2.09	Yes
Adamts5	a disintegrin-like and metallopeptidase (reprolysin type) withthrombospondin type 1 motif, 5 (aggrecanase-2)	0.00287	−2.08	
Kcnj14	potassium inwardly-rectifying channel, subfamily J, member 14	0.00022	−2.07	
Slc16a12	solute carrier family 16 (monocarboxylic acid transporters), member 12	0.00221	−2.07	Yes
Dbi	diazepam binding inhibitor	0.00384	−2.06	Yes
Fasn	fatty acid synthase	0.00220	−2.06	

Batch promoter analysis was performed with Eldorado coupled to matinspector (Genomatix Suite). Yes in the right column indicates the presence in the corresponding gene promoter of a putative PPAR response element (PPRE) with at least 85% homology with the matrix similarity defined by Genomatix.

## Discussion

Here we analyzed the effects of dietary BC on whole-body adiposity and adipose tissue physiology. We supplemented WT and *Bcmo1* ^-/-^ mice with BC and compared them to non-supplemented littermates. Our results revealed a Bcmo1-dependent, adiposity reducing effect of BC supplementation, as percent body fat, adipose depot mass, iWAT adipocyte size and leptin serum levels were all significantly reduced after BC supplementation in the WT mice, but not in the *Bcmo1^-/-^* mice (Figure 3). Reduced adiposity in WT mice after BC supplementation was not associated with adverse effects on blood parameters measured such as increased circulating triacylglycerol levels. Moreover, no harmful effects of BC supplementation on parameters such as body weight gain, food intake, or lung tissue histology were observed in the animals (this work and [38]). Although relatively low dietary vitamin A was fed, blood retinol levels were well maintained and within the expected range for adult mice in the different genotypes and supplementation groups (Figure 1D). Additionally, stored retinol was found in the adipose tissue (Figure 1F) and in the liver (data not shown), indicating no gross changes in vitamin A homeostasis in animals of different supplementation groups.

Microarray analyses revealed that BC supplementation altered the overall iWAT gene expression signature in WT mice but not in *Bcmo1^-/-^* mice. Strikingly, none of the genes altered in its expression levels upon BC supplementation in the WT mice (p<0.01) was changed in *Bcmo1* knockout mice (Figure 4). BC supplementation led to a general down-regulation of gene expression in iWAT of WT mice. This finding indicated that the activity of major transcriptional regulator of iWAT was affected by BC supplementation. Microarray, Q-PCR and immunoblotting analysis consistently confirmed a reduction of PPARγ expression (Figure 5A and 5D). *In silico* analysis of the promoter regions of the top 50 BC down-regulated genes in wild-type mice showed that more than 50% contained a *PPAR* response element (Table 2). The p value associated (1.33e^−6^) strongly supported the importance of PPARγ within this gene set, since it represent the probability to obtain an equal or greater number of sequences with a match in a randomly list of genes. Interestingly, such result was not reproduced in the *Bcmo1* ^-/-^ mice (p value 0.81; not significant) underlying the key role of this enzyme in the repression of PPARγ activity by BC. Thus, the reduction of PPARγ activity and the down-regulation of its target genes likely explain the reduced adiposity of WT mice upon BC supplementation.

Centric cleavage of BC by Bcmo1 leads to retinaldehyde production which can be converted to retinol and retinyl ester for storage and to retinoic acid. Enzymes catalyzing these reactions are expressed in adipocytes [7]; [16]. Retinoids such as retinoic acid and retinaldehyde are ligands for nuclear receptors including RARs and PPARs that help control adipogenesis and adipocyte metabolism. Treatment with retinoic acid has been shown to inhibit differentiation of pre-adipose cells in culture by blocking PPARγ induction in an RAR-mediated manner [18]; [39], and to result *in vivo* in mice in reduced PPARγ expression levels in WAT [19] and reduced whole body adiposity [19]; [31]. Moreover, a recent study showed that BC, but not retinol, decreases PPARγ and reduces lipid storage capacity in mature white adipocytes in culture [15]. This effect involves production of retinoic acid from BC, is blocked by a Bcmo1 chemical inhibitor (fenretinide), and is dependent on RARs [15]. Importantly, BC was accumulated following BC diet in serum and tissues of WT mice (Figure 1C and 1E), suggesting that not all BC undergoes intestinal cleavage in WT mice. Thus, an adipose tissue-specific, Bcmo1-dependent production of retinoids, such as retinoic acid, from BC might explain the down-regulation of iWAT PPARγ expression levels and the overall changes in iWAT gene expression patterns and adiposity-related end-points observed in this work in WT mice following chronic exposure to a BC-enriched diet. The involvement of RAR-signalling is sustained by the *CYP26a1* gene-expression data showing increased expression of this retinoic acid target gene following BC supplementation selectively in the iWAT of WT mice (Figure 5E). Additionally, iWAT mRNA levels of some other genes for which there is evidence of transcriptional up-regulation or down-regulation in response to retinoic acid were significantly increased and decreased, respectively, following BC supplementation in the WT mice, but not in the *Bcmo1*-null mice (microarray data shown in Table S4), including leptin. Leptin expression has been shown to be decreased by retinoic acid [24]–[27] and blood levels of this adipokine were significantly reduced in WT mice supplemented with BC.

Interestingly, expression of the *Bcdo2* gene was up-regulated in iWAT of *Bcmo1*-null mice, both under control and BC diet (Figure 1B). In fact, relatively large amounts of β-10′-apocarotenol became detectable in WAT of *Bcmo1^-/-^* mice upon BC supplementation. This compound derives by the reduction of the primary BC cleavage product β-10′-apo-carotenal. It has been reported that apocarotenoids such as β-14-apo-carotenal can repress PPARγ - and RXR-mediated responses in cell culture [32]. However, we found not much evidence that apocarotenoid formation in *Bcmo1^-/-^* mice influenced adipocyte physiology. Our results indicate that the effects of BC on adipocyte biology are clearly dependent on Bcmo1, as those effects were not found in Bcmo1-deficient animals that express Bcdo2.

In summary, this study revealed a Bcmo1-dependent, adiposity reducing effect of long-term BC supplementation in mice. In this context, we provide evidence that BC-derived retinoids influence adipocyte physiology. *Bcmo1* is a PPARγ target gene [13], and our analysis indicates that the Bcmo1-dependent, adiposity-reducing effect of BC is mediated by a reduction of PPARγ activity in adipocytes. Thus, our findings establish Bcmo1 as key component for the crosstalk between RAR- and PPARγ-signaling pathways in the control of body adiposity.

Our results may also have implications in the context of the current obesity pandemics. There is evidence of an impact of BC and vitamin A status on parameters related to adiposity in humans [40]. Circulating BC levels are inversely correlated with risk of human type 2 diabetes, a pathology associated with obesity [41]–[44] . Additionally, reduced plasma levels of carotenoids, including BC, are commonly found in obese children [45]. Genetic polymorphisms in the *Bcmo1* gene have been described that alter the activity of the encoded human protein [46]; [47]. Our work suggests that BC intake may have different consequences on body adiposity in subjects carrying different Bcmo1 functional gene variants. Thus, the involvement of BC and Bcmo1 in human obesity deserves further research.

## Materials and Methods

### Animals and diets

The animal experiment was conducted according to accepted standards of humane care and use of laboratory animals, and was approved by the bioethical committee of the University of Freiburg (Az.: 35/9185.81/G-07/30). C57/BL6;129Svj-*Bcmo1* ^tm1Dnp^ (*Bcmo1* ^-/-^) mice previously described by Hessel et al. [7] and mixed background F1 generation of C57/BL6;129Svj mice as WT controls were used. During the breeding and weaning periods and up to 5 weeks of age all mice were maintained on breeder chow containing 14000 IU vitamin A/kg diet (Provimi Kliba AG, Kaiseraugst, Switzerland). Five-week-old WT and *Bcmo1* ^-/-^ female mice were fed a defined, pelletized diet containing 1500 IU vitamin A/kg and 10% energy as fat, with (BC diet) or without (control diet) 150 mg BC/kg diet (n = 6 animals per group) for 14 weeks, after which they were euthanized. Detailed composition of these diets is shown in Table 3. A relatively low dose of dietary vitamin A was used to maximize intestinal BC absorption, which can be repressed by preformed vitamin A [48]. The dose used was previously shown to be sufficient to maintain vitamin A homeostasis while allowing tissue BC accumulation both in WT and *Bcmo1* ^-/-^mice [9]. The experimental diets were prepared by Research Diets, Inc (New Brunswick, NJ, USA) by cold extrusion so as to protect BC from heat treatment and incorporating a water-soluble formulation in the form of beadlets containing BC, DL-α-tocopherol, ascorbyl palmitate, corn oil, fish gelatin, sucrose and corn starch. The control diet contained control beadlets devoid of BC, but with all other ingredients. All beadlets were kindly provided by DSM Nutritional Products Ltd (Basel, Switzerland). Mice were maintained under environmentally controlled conditions (24°C, 12 h/12 h light/dark cycle) in groups of 3 animals per cage and with *ad libitum* access to feed and water. Body weight and food intake were determined twice per week (3 and 4 days intervals). Energy intake was estimated on a per-cage basis from the actual amount of food consumed by the animals and its caloric equivalence (16 MJ/kg for both control and BC diet). On the day of the necropsy, the animals were sacrificed according to the animal protocol of the University of Freiburg (Germany). First, animals were subjected to inhalation of isofluran. Then, 100–150 mg/kg KGW Ketamin (Ketamin 10%, Essex) and 5–10 mg/kg KGW Xylazin (Rompun 2 %, Bayer) were injected intraperitoneally. Blood was drawn from the vena cava, and animals were then sacrificed by cervical dislocation. Inguinal (iWAT), gonadal (periovarian) and retroperitoneal white adipose tissue (WAT) depots were excised in their entirety according to Cinti [49], weighed, snap-frozen in liquid nitrogen and stored at –80°C until analysis. The continuous subcutaneous fat depot comprising the dorsolumbar and inguinal areas was taken as iWAT. The combined mass of all WAT pads taken expressed as a percentage of body weight was used as adiposity index. For three animals in each group, one entire iWAT lobule was fixed for histological studies.

**Table 3:** Composition of the control and beta-carotene (BC) diets.

	Control diet	BC diet
Macronutrients		
Protein (g/100 g)	19.2	19.2
Carbohydrate (g/100 g)	67.2	67.2
Fat (g/100 g)	4.3	4.3
Kcal/g (MJ/kg)	3.84 (16)	3.84 (16)
Calories from protein (%)	20	20
Calories from carbohydrate (%)	70	70
Calories from fat (%)	10	10
Ingredients (g/Kg)		
Casein, 30 mesh	189	189
L-Cystine	2.8	2.8
Corn Starch	298	298
Maltodextrin 10	33	33
Sucrose	331	331
Cellulose, BW200	47	47
Soybean Oil	24	24
Lard	19	19
Salt Mix, S10026	9.5	9.5
DiCalcium Phosphate	12	12
Calcium Carbonate	5.2	5.2
Potassium Citrate, 1 H2O	16	16
Vitamin Mix V13001 with no added vitamin A	9.5	9.5
Vitamin A (retinol acetate, 500,000 IU/g)	0.003	0.003
Choline bitartrate	1.9	1.9
BC beadlets with 100 mg BC/g*	0	1.5
Control beadlets without BC	1.5	0
FD&C Yellow Dye #5	0	0.024
FD&C Red Dye #40	0.047	0
FD&C Blue Dye #1	0	0.024

BC beadlets contained BC, DL-α-tocopherol, ascorbyl palmitate, corn oil, fish gelatin, sucrose and corn starch. Control beadlets were devoid of BC, but with all other ingredients. All beadlets were kindly provided by DSM Nutritional Products Ltd (Basel, Switzerland). The experimental diets were prepared by Research Diets, Inc (New Brunswick, NJ, USA) by cold extrusion so as to protect BC from heat treatment.

### Histology and immunohistochemistry

Entire iWAT lobules were fixed by immersion in the perfusion fixative (4% paraformaldehyde in 0.1 M sodium phosphate buffer, pH 7.4) overnight at 4°C, dehydrated, cleared and then paraffin-embedded so that the plane of section corresponded with the one of the wider surface. 3 µm-thick sections at the same level were obtained and stained with hematoxylin/eosin for morphometric analysis, performed by digital acquisition of adipose tissue areas (Digital Still Camera DXM 1200 and NIKON 6000 Eclipse Microscope). Two histological sections were counted per mouse.

### Serum parameters

Commercial enzymatic colorimetric kits were used for the determination of circulating levels of triacylglycerol (Triglyceride (INT) 20, Sigma Diagnostics, St Louis, MO, USA) and glucose (D-Glucose UV-method, Roche Biopharm GmbH, Darmstadt, Germany). Serum levels of leptin and insulin were measured as described before [50] with some minor differences. Briefly, sera were diluted 4x in High Performance ELISA buffer (Sanquin, Amsterdam, The Netherlands) before adipokine analyses using the mouse serum adipokine Lincoplex Kit (Linco Research, Nuclilab, Ede, The Netherlands). The assays were conducted according to the manufacturer's protocol and measured using the Bio-PLex (Bio-Rad, Venendaal, The Netherlands) system with Bio-Plex software. All individual samples were analyzed in duplicate and averaged.

### Analysis of retinoids, apocarotenoids, and carotenoids

Retinoids and carotenoids were extracted from serum under dim red safety light (600 nm) as described previously [7]. Adipose tissue (∼40 mg) was incubated with 100 µL of 12% pyrogallol in ethanol, 200 µL of 30% KOH in water and 1 ml ethanol for 2 h at 37°C. After saponification, 1 ml H_2_O was added. The samples were extracted with 3 ml of ether/hexane (2∶1, stabilized with 1% ethanol) twice, and diluted with 2 ml H_2_O and 2 ml ethanol. After centrifugation for 5 min at 800× *g*, the organic layer was collected. The extract was evaporated in a Speedvac and dissolved in HPLC solvent. HPLC separation of carotenoids and retinoids and quantification of peak integrals was performed as described previously [6]. Solvents for HPLC and extraction were purchased in HPLC grade from Merck (Darmstadt, Germany). β-10′-apocarotenal was a gift of Dr. Hansgeorg Ernst (BASF, Germany). For β-10′-apocarotenol production, the standard was dissolved in 1 ml ethanol and reduced with BH_4_ on ice. After 20 min, reaction products were extracted upon addition of 1 ml H_2_O with 1 ml hexanes. The organic phase was dried with a speedvak and redissolved in hexanes and subjected to HPLC analysis. The identity of β-10′-apocarotenol was further confirmed by LC/MS. The saponified lipophilic extract from iWAT was injected onto a Zorbax Sil column (Agilent Technologies, Santa Clara, CA) equilibrated with 90 % hexane and 10 % acetylacetate (v:v). The eluant was directed into LXQ mass spectrometer through an atmospheric pressure chemical ionization source (Thermo Scientific Waltham, MA) working in a positive mode. β-10′-apocarotenol was identified based on its molecular mass (m/z  = 361.42 [MH]^+^) and a fragmentation pattern of the parent ion identical with that of the β-10′-apocarotenol standard.

### RNA isolation

WAT was homogenized in liquid nitrogen using a liquid nitrogen cooled mortar and pestle. Total RNA was isolated using TRIzol reagent (Invitrogen, Carlsbad, CA, USA) followed by purification using RNeasy columns (Qiagen, Venlo, The Netherlands) according to the instructions of the manufacturer. RNA concentration and purity were measured using the Nanodrop spectrophotometer (ND-1000, Isogen Life Science, Maarssen, The Netherlands). Approximately 20 µg of total RNA was isolated from each WAT depot. A260/A280 ratios were above 2 and A260/A230 ratios were above 1.9 for all samples, indicating good RNA purity. RNA degradation was checked using StdSense Chips (Biorad) and no apparent RNA degradation was seen.

### Q-PCR analysis

cDNA was prepared by reverse transcription of 1 µg total RNA using random hexamers as primers with M-MLV reverse transcriptase (Invitrogen). The expression of selected cDNAs was analyzed by real-time quantitative PCR, as reported previously [51]. Reactions were performed in duplicate with a Stratagene MX3005P apparatus (Stratagene, Amsterdam, The Netherlands) using SYBR green kit (Eurogentec, Angers, France), according to the manufacturer instructions. Primers used were as follows: for PPARγ, 5′-CAA GAA TAC CAA AGT GCG ATC AA-3′ and 5′-GAG CTG GGT CTT TTC AGA ATA ATA AG-3′; for RXRα, 5′-GCC ATC TTT GAC AGG GTG CTA-3′ and 5′-CTC CGT CTT GTC CAT CTG CAT-3′; for lipoprotein lipase (LPL), 5′-GGC CAG ATT CAT CAA CTG GAT-3′ and 5′-GCT CCA AGG CTG TAC CCT AAG-3′; for β-carotene 9′, 10′-dioxygenase (Bcdo2), 5′-CCT GGT GAG TAT TCC CTC ACA-3′ and 5′-TCT CAA CTG TTTCTG CGT TTC-3′; and for Bcmo1, 5′-TCT GAG TTC GGA ACC ATG GC-3′ and 5′-GTG TGA GAC AAG TAG GAG AAA GCT-3′. The relative levels of mRNA were calculated using the comparative ΔΔCt method. 18S rRNA (5′- CGC CGC TAG AGG TGA AAT TCT-3′ and 5′- CAT TCT TGG CAA ATG CTT TCG-3′) was used as reference gene. For CYP26a1 mRNA quantification, the TaqMan Gene Expression Assay Mm00514486-s1 was used together with the assay Mm00507222-s1 for 18S rRNA (Applied Biosystems, Courtaboeuf, France).

### Western blot analysis

For PPARγ determination in iWAT, 100 µg of total protein from each animal tissue was fractionated in SDS-polyacrylamide (12% acrylamide) and then electroblotted to a PVDF membrane. Membranes were blocked in fat-free milk (5%) dissolved in Tris buffered saline-Tween, washed and incubated overnight at 4°C with anti-mouse PPARγ (Abcam, Cambridge, MA) diluted 1∶500. As a protein loading control, β-actin (Cell Signalling, Boston, MA) diluted 1∶1000 was used. Secondary antibody (1∶5000) was horseradish peroxidase-conjugated anti-rabbit IgG (Promega, Madison, WI). Immunoblots were developed with the ECL system (Amersham Biosciences) and scanned. Quantification of bands was performed by the ImageJ (Java).

### Microarray hybridization procedure

The 4 x 44 k Agilent whole mouse genome microarrays (G4122F, Agilent Technologies, Inc. Santa Clara, CA, USA) were used and microarray hybridization was performed essentially according to the manufacturer's protocol. In brief, cDNA was synthesized out of 1 µg iWAT RNA using the Agilent Low RNA Input Fluorescent Linear Amplification Kit for each animal without addition of spikes. Thereafter samples were split in 2 equal amounts, to synthesize Cyanine 3-CTP (Cy3) and Cyanine 5-CTP (Cy5) labeled cRNA using half the amounts as indicated by the manufacturer per dye (Agilent Technologies) and as has been assessed previously [50]. Labeled cRNA was purified using RNeasy columns (Qiagen). Yield, A260/A280 ratio and Cy3 or Cy5 activity were examined for every sample on the nanodrop. All samples met the criteria of a cRNA yield higher than 825 ng and a specific activity of at least 8.0 pmol Cy3 or Cy5. 1200 ng of every Cy3 labeled cRNA sample was pooled and used as a common reference pool. Individual 825 ng Cy5-labeled cRNA and 825 ng pooled Cy3-labeled cRNA were fragmented in 1x fragmentation and 1x blocking agent (Agilent Technologies) at 60°C for 30 minutes and thereafter mixed with GEx Hybridization Buffer HI-RPM (Agilent Technologies) and hybridized in a 1∶1 ratio at 65°C for 17 h in an Agilent Microarray hybridization chamber rotating at 4 rpm. After hybridization slides were washed according to the wash protocol with Stabilization and Drying solution (Agilent Technologies). Arrays were scanned at an Agilent scanner with 10% and 100% laser power intensities (Agilent Technologies).

### Data analyses and statistical methods

Signal intensities for each microarray spot were quantified using Feature Extraction 9.1 (Agilent Technologies). Median density values and background values of each spot were extracted for both the experimental samples (Cy5) and the reference samples (Cy3). Quality control was performed visually, by using Quality control graphs from Feature extraction and M-A plots and boxplots were made using limmaGUI in R (Bioconductor) [52] for every microarray. Data were imported into GeneMaths XT (Applied Maths, Sint-Martens-Latem, Belgium). Spots with a Cy5 and Cy3 signal twice above background were selected and log transformed. 31295 spots had at least a 2-fold signal to noise ratio. The Cy5 signal was normalized against the Cy3 intensity as described before [53]. P-values for differential expressions were calculated between 2 groups using t-test statistics on log intensity values. Changes were considered statistically different at p<0.05. Vulcano plots were made using Graphpad Prism 5.0.2 (La Jolla, CA) with all spots or a selection of spots. Heatmapping and hierarchical clustering was performed in GeneMathsXT (Applied Maths, St.Martens Lathem, Belgium). Pathway analysis was performed using MetaCore (GeneGo Inc, St. Joseph, MI, USA). Promoter analysis and the literature based network were performed with the Genomatix suite (Genomatix Software GmbH, Germany). Biochemical and biometric parameters in Figures 1, 2, and 4 are presented as means ± SEM. Statistical significance of effects on these parameters was assessed by two-way ANOVA and two-tailed Student's *t* test using SPSS 14.0 for windows (SPSS, Chicago, IL, USA), with threshold of significance set at p<0.05. Microarray data has been submitted in a MIAME compliant form to Gene Expression Omnibus (GEO) data base of the National Center of Biotechnology Information (NCBI),, and is accessible through GEO series accession number GSE27271 (http://www.ncbi.nlm.nih.gov/geo/query/acc.cgi?acc=GSE27271).

## Supporting Information

Figure S1 (**A**) *PPARγ* mRNA levels, (**B**) *RXRα* mRNA levels, (**C**) *lipoprotein lipase* (*LPL*) mRNA levels, (**D**) *Bcmo1* mRNA levels and (**E**) *Bcdo2* mRNA levels in gonadal white adipose tissue of wild-type (WT) and *Bcmo1*-null (*Bcmo1* ^-/-^) mice after 14 weeks on a control diet (open bars) or a β-carotene -enriched diet (black bars). Quantitative real-time PCR was used to determine normalized gene expression levels as described in Materials and Methods. Data are the mean ± SEM of 6 animals per group. G, effect of genotype in two-way ANOVA analysis (p<0.05).
(TIF)

Table S1 **Genes differentially regulated (p<0.01) in white adipose tissue of Bcmo1-/- mice after β-carotene supplementation.**
(DOC)

Table S2 **Main pathways affected by dietary β-carotene supplementation in inguinal white adipose tissue of wild-type mice as indicated by Metacore analysis.** Differentially expressed genes at p<0.05 involved in aspects of metabolism as indicated are shown.
(DOC)

Table S3 **Changes (p<0.05) in the expression of angiogenesis-related genes in inguinal white adipose tissue of wild-type and** ***Bcmo1*** **-null mice after 14 weeks of dietary β-carotene supplementation.**
(DOC)

Table S4 **Beta-carotene Reduces Body Adiposity of Mice via BCMO1.**
(DOC)
