# Supplementary material for: Beta-Carotene Reduces Body Adiposity of Mice via BCMO1
Source: PLoS One. 2011 Jun 1;6(6):e20644. doi: 10.1371/journal.pone.0020644 (PMC3106009; doi:10.1371/journal.pone.0020644)
Supplement: Table S1 — Genes differentially regulated (p<0.01) in white adipose tissue of Bcmo1-/- mice after β-carotene supplementation. (DOC) [file pone.0020644.s002.doc]

**Supplementary Information**

**Beta-carotene Reduces Body Adiposity of Mice via BCMO1**

Jaume Amengual, Erwan Gouranton, Yvonne G. J. van Helden, Susanne Hessel, Joan Ribot, Evelien Kramer, Beata Kiec-Wilk, Ursula Razny, Georg Lietz, Adrian Wyss, Aldona Dembinska-Kiec, Andreu Palou, Jaap Keijer, Jean François Landrier, M. Luisa Bonet^#^ and Johannes von Lintig^#^

# Corresponding authors: M. Luisa Bonet, Laboratory of Molecular Biology, Nutrition and Biotechnology. Department of Fundamental Biology and Health Sciences, Universitat de les Illes Balears, Crta. Valldemossa Km 7.5, 07122, Palma de Mallorca, Spain. E-mail: luisabonet@uib.es and Johannes von Lintig, Department of Pharmacology, School of Medicine, Case Western Reserve University, Cleveland, OH, USA. E-mail: johannes.vonlintig@case.edu.

**Supplementary table 1. Genes differentially regulated (p<0.01) in white adipose tissue of *Bcmo1*^-/-^ mice after β-carotene supplementation**

|  |  |  |  |
| --- | --- | --- | --- |
| **Gene** | **Gene name** | **p value** | **fold-change** |
| 1110065P20Rik | RIKEN cDNA 1110065P20 gene | 0.00618 | -1.14 |
| 1600002H07Rik | RIKEN cDNA 1600002H07 gene | 0.00791 | 1.14 |
| 1700018G05Rik | RIKEN cDNA 1700018G05 gene | 0.00235 | 2.33 |
| Slc22a15 | RIKEN cDNA 2610034P21 gene | 0.00739 | -1.13 |
| Tmem178 | RIKEN cDNA 2810417M05 gene | 0.00850 | 1.36 |
| 3830406C13Rik | RIKEN cDNA 3830406C13 gene | 0.00698 | -1.16 |
| Lyrm1 | RIKEN cDNA 4930404J24 gene | 0.00943 | -1.24 |
| 4930581F22Rik | RIKEN cDNA 4930581F22 gene | 0.00893 | -1.22 |
| 4932442K08Rik | RIKEN cDNA 4932442K08 gene | 0.00422 | -1.18 |
| 6530401N04Rik | RIKEN cDNA 6530401N04 gene | 0.00201 | -1.21 |
| 9530077C05Rik | RIKEN cDNA 9530077C05 gene | 0.00995 | -1.19 |
| Gbp6 | RIKEN cDNA 9830147J24 gene | 0.00431 | -1.26 |
| Abcc5 | ATP-binding cassette, sub-family C (CFTR/MRP), member 5 | 0.00534 | -1.33 |
| Acot11 | acyl-CoA thioesterase 11 | 0.00204 | 2.72 |
| Adprhl1 | ADP-ribosylhydrolase like 1 | 0.00990 | 1.91 |
| Agtr1a | angiotensin II receptor, type 1a | 0.00633 | 1.57 |
| Ankrd50 | ankrin repeat domain 50 | 0.00934 | -1.19 |
| Arih1 | ariadne ubiquitin-conjugating enzyme E2 binding protein homolog 1 (Drosophila) | 0.00486 | 1.16 |
| B230311B06Rik | RIKEN cDNA B230311B06 gene | 0.00901 | 4.14 |
| B430119L13Rik | RIKEN cDNA B430119L13 gene | 0.00132 | -1.50 |
| Vprbp | RIKEN cDNA B930007L02 gene | 0.00404 | -1.16 |
| BC013672 | cDNA sequence BC013672 | 0.00612 | -1.59 |
| BC020489 | cDNA sequence BC020489 | 0.00478 | -1.86 |
| BC049806 | cDNA sequence BC049806 | 0.00994 | -1.28 |
| Chd9 | chromodomain helicase DNA binding protein 9 | 0.00981 | -1.13 |
| Coq7 | demethyl-Q 7 | 0.00694 | 1.67 |
| Reep3 | DNA segment, Chr 10, University of California at Los Angeles 1 | 0.00682 | -1.20 |
| Heatr5a | RIKEN cDNA D930036F22 gene | 0.00776 | -1.25 |
| Clpx | DNA segment, Chr 9, ERATO Doi 338, expressed | 0.00332 | -1.27 |
| Dctn6 | dynactin 6 | 0.00121 | -1.10 |
| Dcun1d1 | DCUN1D1 DCN1, defective in cullin neddylation 1, domain containing 1 (S. cerevisiae) | 0.00312 | 1.09 |
| E030041M21Rik | RIKEN cDNA E030041M21 gene | 0.00813 | -1.34 |
| Edd1 | E3 ubiquitin protein ligase, HECT domain containing, 1 | 0.00504 | -1.17 |
| Egfl9 | EGF-like-domain, multiple 9 | 0.00398 | 1.26 |
| Eltd1 | EGF, latrophilin seven transmembrane domain containing 1 | 0.00625 | -1.23 |
| Fgf10 | fibroblast growth factor 10 | 0.00526 | -1.38 |
| Grn | Granulin | 0.00726 | -1.23 |
| Gucy1a3 | guanylate cyclase 1, soluble, alpha 3 | 0.00103 | -1.23 |
| Hmgb2l1 | high mobility group box 2-like 1 | 0.00341 | 1.10 |
| Ifih1 | interferon induced with helicase C domain 1 | 0.00546 | -1.32 |
| Itgb3bp | integrin beta 3 binding protein (beta3-endonexin) | 0.00807 | -1.11 |
| Itm2b | integral membrane protein 2B | 0.00440 | -1.19 |
| Las1l | LAS1-like (S. cerevisiae) | 0.00477 | -1.09 |
| LOC627914 | similar to gonadotropin inducible ovarian transcription factor 1 | 0.00763 | -1.18 |
| Mthfs | 5, 10-methenyltetrahydrofolate synthetase | 0.00422 | -1.08 |
| Nnmt | nicotinamide N-methyltransferase | 0.00154 | -1.80 |
| Nup155 | nucleoporin 155 | 0.00252 | 1.51 |
| Oas2 | 2'-5' oligoadenylate synthetase 2 | 0.00614 | -1.49 |
| Opn3 | opsin (encephalopsin) | 0.00449 | -1.56 |
| P2ry6 | pyrimidinergic receptor P2Y, G-protein coupled, 6 | 0.00013 | -1.22 |
| Parp9 | poly (ADP-ribose) polymerase family, member 9 | 0.00614 | -1.19 |
| Pcf11 | cleavage and polyadenylation factor subunit homolog (S. cerevisiae) | 0.00093 | -1.11 |
| Pdgfra | platelet derived growth factor receptor, alpha polypeptide | 0.00356 | -1.15 |
| Pdia4 | protein disulfide isomerase associated 4 | 0.00547 | -1.18 |
| Pold3 | polymerase (DNA-directed), delta 3, accessory subunit | 0.00839 | 1.84 |
| Rab6 | RAB6, member RAS oncogene family | 0.00179 | 1.62 |
| Rbm4 | RNA binding motif protein 4 | 0.00578 | 1.16 |
| Skap2 | src family associated phosphoprotein 2 | 0.00796 | -1.14 |
| Serpina3k | serine (or cysteine) peptidase inhibitor, clade A, member 3K | 0.00241 | -1.45 |
| Slc1a3 | solute carrier family 1 (glial high affinity glutamate transporter), member 3 | 0.00133 | -2.07 |
| Slc30a9 | solute carrier family 30 (zinc transporter), member 9 | 0.00081 | -1.20 |
| Sp100 | nuclear antigen Sp100 | 0.00797 | -1.39 |
| St6galnac5 | ST6 (alpha-N-acetyl-neuraminyl-2,3-beta-galactosyl-1,3)-N-acetylgalactosaminide alpha-2,6-sialyltransferase 5 | 0.00362 | -1.80 |
| Stab1 | stabilin 1 | 0.00926 | -1.21 |
| Stat2 | signal transducer and activator of transcription 2 | 0.00460 | -1.37 |
| Tfb1m | transcription factor B1, mitochondrial | 0.00557 | -1.13 |
| Tmem18 | transmembrane protein 18 | 0.00787 | -1.13 |
| Trim25 | tripartite motif protein 25 | 0.00917 | -1.24 |
| Ucp3 | uncoupling protein 3 (mitochondrial, proton carrier) | 0.00465 | 2.14 |
| Usp40 | ubiquitin specific peptidase 40 | 0.00366 | -1.16 |
| Zfp275 | zinc finger protein 275 | 0.00051 | -1.24 |
| Zfp508 | zinc finger protein 508 | 0.00324 | -1.13 |
| D430007A19Rik | zinc finger protein 608 | 0.00677 | -1.40 |
| Zfp72 | zinc finger protein 72 | 0.00461 | -1.12 |
| Zfp74 | zinc finger protein 74 | 0.00199 | -1.17 |
| BC056481 |  | 0.00485 | -1.2 |
| NAP046281-1 |  | 0.00164 | -2.2 |
| AK042238 |  | 0.00988 | -1.2 |
| AK037544 |  | 0.00500 | -1.1 |
| NAP040605-1 |  | 0.00845 | -1.3 |
| TC1651824 |  | 0.00959 | -1.5 |
