# Supplementary material for: Beta-Carotene Reduces Body Adiposity of Mice via BCMO1
Source: PLoS One. 2011 Jun 1;6(6):e20644. doi: 10.1371/journal.pone.0020644 (PMC3106009; doi:10.1371/journal.pone.0020644)
Supplement: Table S2 — Main pathways affected by dietary β-carotene supplementation in inguinal white adipose tissue of wild-type mice as indicated by Metacore analysis. Differentially expressed genes at p<0.05 involved in aspects of metabolism as indicated are shown. (DOC) [file pone.0020644.s003.doc]

**Supplementary Information**

**Beta-carotene Reduces Body Adiposity of Mice via BCMO1**

Jaume Amengual, Erwan Gouranton, Yvonne G. J. van Helden, Susanne Hessel, Joan Ribot, Evelien Kramer, Beata Kiec-Wilk, Ursula Razny, Georg Lietz, Adrian Wyss, Aldona Dembinska-Kiec, Andreu Palou, Jaap Keijer, Jean François Landrier, M. Luisa Bonet^#^ and Johannes von Lintig^#^

# Corresponding authors: M. Luisa Bonet, Laboratory of Molecular Biology, Nutrition and Biotechnology. Department of Fundamental Biology and Health Sciences, Universitat de les Illes Balears, Crta. Valldemossa Km 7.5, 07122, Palma de Mallorca, Spain. E-mail: luisabonet@uib.es and Johannes von Lintig, Department of Pharmacology, School of Medicine, Case Western Reserve University, Cleveland, OH, USA. E-mail: johannes.vonlintig@case.edu.

**Supplementary Table 2. Main pathways affected by dietary β-carotene supplementation in inguinal white adipose tissue of wild-type mice as indicated by Metacore analysis.** Differentially expressed genes at p<0.05 involved in aspects of metabolism as indicated are shown.

| **Gene** | **Gene name** | **p value** | **fold-change** |
| --- | --- | --- | --- |
| **Oxidative phosphorylation** | |  |  |
| [ATP5A1](http://lingenego.voeding.tno.nl:8000/cgi/gene.cgi?id=-1419140076) | ATP synthase subunit alpha, mitochondrial precursor | 0.00749 | -1.23 |
| [ATP5B](http://lingenego.voeding.tno.nl:8000/cgi/gene.cgi?id=1596673920) | ATP synthase subunit beta, mitochondrial precursor | 0.02293 | -1.41 |
| [ATP5C1](http://lingenego.voeding.tno.nl:8000/cgi/gene.cgi?id=736350337) | ATP synthase subunit gamma, mitochondrial precursor | 0.04245 | -1.23 |
| Atp5e | ATP synthase, H+ transporting, mitochondrial F1 complex, epsilon subunit | 0.03268 | -1.40 |
| [ATP5F1](http://lingenego.voeding.tno.nl:8000/cgi/gene.cgi?id=2042645043) | ATP synthase subunit b, mitochondrial precursor | 0.00263 | -1.29 |
| [ATP5G3](http://lingenego.voeding.tno.nl:8000/cgi/gene.cgi?id=-2020760740) | ATP synthase lipid-binding protein, mitochondrial precursor | 0.02206 | -1.61 |
| Atp5h | ATP synthase, H+ transporting, mitochondrial F0 complex, subunit d | 0.01274 | -1.34 |
| [ATP5J](http://lingenego.voeding.tno.nl:8000/cgi/gene.cgi?id=-1789067356) | ATP synthase-coupling factor 6, mitochondrial precursor | 0.00562 | -1.35 |
| Atp5o | ATP synthase, H+ transporting, mitochondrial F1 complex, O subunit | 0.02142 | -1.32 |
| Atp6v0a1 | ATPase, H+ transporting, lysosomal V0 subunit A1 | 0.00385 | -1.76 |
| Atp6v0e2 | ATPase, H+ transporting, lysosomal V0 subunit E2 | 0.01174 | -1.55 |
| Atp6v1a | ATPase, H+ transporting, lysosomal V1 subunit A | 0.02200 | -1.21 |
| Atp6v1b2 | ATPase, H+ transporting, lysosomal V1 subunit B2 | 0.02885 | -1.08 |
| Atp6v1d | ATPase, H+ transporting, lysosomal V1 subunit D | 0.02110 | -1.20 |
| Atp6v1f | ATPase, H+ transporting, lysosomal V1 subunit F | 0.01636 | -1.09 |
| Atp6v1h | ATPase, H+ transporting, lysosomal V1 subunit H | 0.00828 | -1.33 |
| Cox11 | COX11 homolog, cytochrome c oxidase assembly protein (yeast) | 0.00678 | -1.21 |
| Cox15 | COX15 homolog, cytochrome c oxidase assembly protein (yeast) | 0.01506 | -1.38 |
| [COX4I1](http://lingenego.voeding.tno.nl:8000/cgi/gene.cgi?id=-880257653) | Cytochrome c oxidase subunit 4 isoform 1, mitochondrial precursor | 0.04872 | -1.27 |
| [COX5B](http://lingenego.voeding.tno.nl:8000/cgi/gene.cgi?id=1197728517) | Cytochrome c oxidase subunit 5B, mitochondrial precursor | 0.02493 | -1.28 |
| [COX6A1](http://lingenego.voeding.tno.nl:8000/cgi/gene.cgi?id=-223031063) | Cytochrome c oxidase polypeptide VIa-liver, mitochondrial precursor | 0.01568 | -1.42 |
| [COX8A](http://lingenego.voeding.tno.nl:8000/cgi/gene.cgi?id=-208079536) | Cytochrome c oxidase polypeptide VIII-liver/heart, mitochondrial precursor | 0.00724 | -1.52 |
| [CYCS](http://lingenego.voeding.tno.nl:8000/cgi/gene.cgi?id=1757475701) | Cytochrome c | 0.00419 | -1.39 |
| [NDUFA12](http://lingenego.voeding.tno.nl:8000/cgi/gene.cgi?id=1946917576) | NADH dehydrogenase [ubiquinone] 1 alpha subcomplex subunit 12 | 0.00174 | -1.47 |
| [NDUFA13](http://lingenego.voeding.tno.nl:8000/cgi/gene.cgi?id=-1466168244) | NADH dehydrogenase [ubiquinone] 1 alpha subcomplex subunit 13 | 0.02935 | -1.45 |
| [NDUFA2](http://lingenego.voeding.tno.nl:8000/cgi/gene.cgi?id=-531560616) | NADH dehydrogenase [ubiquinone] 1 alpha subcomplex subunit 2 | 0.03388 | -1.55 |
| [NDUFA3](http://lingenego.voeding.tno.nl:8000/cgi/gene.cgi?id=-479821023) | NADH dehydrogenase [ubiquinone] 1 alpha subcomplex subunit 3 | 0.02426 | -1.32 |
| [NDUFA4](http://lingenego.voeding.tno.nl:8000/cgi/gene.cgi?id=1221933774) | NADH dehydrogenase [ubiquinone] 1 alpha subcomplex subunit 4 | 0.01976 | -1.42 |
| [NDUFA5](http://lingenego.voeding.tno.nl:8000/cgi/gene.cgi?id=1420654717) | NADH dehydrogenase [ubiquinone] 1 alpha subcomplex subunit 5 | 0.02918 | -1.30 |
| [NDUFA6](http://lingenego.voeding.tno.nl:8000/cgi/gene.cgi?id=2064737831) | NADH dehydrogenase [ubiquinone] 1 alpha subcomplex subunit 6 | 0.03161 | -1.28 |
| [NDUFA8](http://lingenego.voeding.tno.nl:8000/cgi/gene.cgi?id=53075104) | NADH dehydrogenase [ubiquinone] 1 alpha subcomplex subunit 8 | 0.02034 | -1.31 |
| [NDUFA9](http://lingenego.voeding.tno.nl:8000/cgi/gene.cgi?id=-2139195824) | NADH dehydrogenase [ubiquinone] 1 alpha subcomplex subunit 9, mitochondrial precursor | 0.01213 | -1.33 |
| Ndufab1 | NADH dehydrogenase (ubiquinone) 1. alpha/beta subcomplex, 1 | 0.03112 | -1.29 |
| [NDUFB10](http://lingenego.voeding.tno.nl:8000/cgi/gene.cgi?id=-485735275) | NADH dehydrogenase [ubiquinone] 1 beta subcomplex subunit 10 | 0.03429 | -1.40 |
| [NDUFB11](http://lingenego.voeding.tno.nl:8000/cgi/gene.cgi?id=54539) | NADH dehydrogenase [ubiquinone] 1 beta subcomplex subunit 11, mitochondrial precursor | 0.04603 | -1.22 |
| [NDUFB2](http://lingenego.voeding.tno.nl:8000/cgi/gene.cgi?id=813784547) | NADH dehydrogenase [ubiquinone] 1 beta subcomplex subunit 2, mitochondrial precursor | 0.01125 | -1.43 |
| [NDUFB3](http://lingenego.voeding.tno.nl:8000/cgi/gene.cgi?id=-118279932) | NADH dehydrogenase [ubiquinone] 1 beta subcomplex subunit 3 | 0.04032 | -1.46 |
| [NDUFB5](http://lingenego.voeding.tno.nl:8000/cgi/gene.cgi?id=-1733446421) | NADH dehydrogenase [ubiquinone] 1 beta subcomplex subunit 5, mitochondrial precursor | 0.00654 | -1.31 |
| [NDUFB6](http://lingenego.voeding.tno.nl:8000/cgi/gene.cgi?id=1615893183) | NADH dehydrogenase [ubiquinone] 1 beta subcomplex subunit 6 | 0.00691 | -1.43 |
| [NDUFB7](http://lingenego.voeding.tno.nl:8000/cgi/gene.cgi?id=959103676) | NADH dehydrogenase [ubiquinone] 1 beta subcomplex subunit 7 | 0.03653 | -1.40 |
| [NDUFB8](http://lingenego.voeding.tno.nl:8000/cgi/gene.cgi?id=1574363795) | NADH dehydrogenase [ubiquinone] 1 beta subcomplex subunit 8, mitochondrial precursor | 0.01714 | -1.37 |
| [NDUFB9](http://lingenego.voeding.tno.nl:8000/cgi/gene.cgi?id=1764906316) | NADH dehydrogenase [ubiquinone] 1 beta subcomplex subunit 9 | 0.01119 | -1.47 |
| [NDUFC1](http://lingenego.voeding.tno.nl:8000/cgi/gene.cgi?id=640738683) | NADH dehydrogenase [ubiquinone] 1 subunit C1. mitochondrial precursor | 0.03569 | -1.28 |
| [NDUFS1](http://lingenego.voeding.tno.nl:8000/cgi/gene.cgi?id=1841903029) | NADH-ubiquinone oxidoreductase 75 kDa subunit, mitochondrial precursor | 0.01817 | -1.31 |
| [NDUFS4](http://lingenego.voeding.tno.nl:8000/cgi/gene.cgi?id=320914780) | NADH dehydrogenase [ubiquinone] iron-sulfur protein 4, mitochondrial precursor | 0.00360 | -1.46 |
| [NDUFS5](http://lingenego.voeding.tno.nl:8000/cgi/gene.cgi?id=-677320856) | NADH dehydrogenase [ubiquinone] iron-sulfur protein 5 | 0.00919 | -1.59 |
| [NDUFS6](http://lingenego.voeding.tno.nl:8000/cgi/gene.cgi?id=1357588599) | NADH dehydrogenase [ubiquinone] iron-sulfur protein 6, mitochondrial precursor | 0.03894 | -1.22 |
| [NDUFV3](http://lingenego.voeding.tno.nl:8000/cgi/gene.cgi?id=808842711) | NADH dehydrogenase [ubiquinone] flavoprotein 3, mitochondrial precursor | 0.03148 | -1.45 |
| Ppa2 | pyrophosphatase (inorganic) 2 | 0.00941 | -1.25 |
| [SDHA](http://lingenego.voeding.tno.nl:8000/cgi/gene.cgi?id=945249494) | Succinate dehydrogenase [ubiquinone] flavoprotein subunit, mitochondrial precursor | 0.02661 | -1.23 |
| [SDHB](http://lingenego.voeding.tno.nl:8000/cgi/gene.cgi?id=1658142531) | Succinate dehydrogenase [ubiquinone] iron-sulfur subunit, mitochondrial precursor | 0.03517 | -1.27 |
| [SDHC](http://lingenego.voeding.tno.nl:8000/cgi/gene.cgi?id=-926185187) | Succinate dehydrogenase cytochrome b560 subunit, mitochondrial precursor | 0.01218 | -1.29 |
| [SDHD](http://lingenego.voeding.tno.nl:8000/cgi/gene.cgi?id=-2029351914) | Succinate dehydrogenase [ubiquinone] cytochrome b small subunit, mitochondrial precursor | 0.00960 | -1.46 |
| Tcirg1 | T-cell, immune regulator 1. ATPase, H+ transporting, lysosomal V0 protein A3 | 0.00810 | 1.32 |
| [UCRC](http://lingenego.voeding.tno.nl:8000/cgi/gene.cgi?id=29796) | Cytochrome b-c1 complex subunit 9 | 0.04344 | -1.34 |
| [UQCR](http://lingenego.voeding.tno.nl:8000/cgi/gene.cgi?id=1144367032) | Cytochrome b-c1 complex subunit 10 | 0.03619 | -1.42 |
| Uqcrb | ubiquinol-cytochrome c reductase binding protein | 0.03401 | -1.30 |
| [UQCRC1](http://lingenego.voeding.tno.nl:8000/cgi/gene.cgi?id=2056008548) | Cytochrome b-c1 complex subunit 1, mitochondrial precursor | 0.01805 | -1.36 |
| [UQCRC2](http://lingenego.voeding.tno.nl:8000/cgi/gene.cgi?id=-973169160) | Cytochrome b-c1 complex subunit 2, mitochondrial precursor | 0.04291 | -1.23 |
| [UQCRFS1](http://lingenego.voeding.tno.nl:8000/cgi/gene.cgi?id=897111986) | Cytochrome b-c1 complex subunit Rieske, mitochondrial precursor | 0.03661 | -1.24 |
| Uqcrh | ubiquinol-cytochrome c reductase hinge protein | 0.04311 | -1.25 |
| [UQCRQ](http://lingenego.voeding.tno.nl:8000/cgi/gene.cgi?id=-593864708) | Cytochrome b-c1 complex subunit 8 | 0.03255 | -1.36 |
| **Regulation of lipid metabolism** | |  |  |
| [ACACA](http://lingenego.voeding.tno.nl:8000/cgi/gene.cgi?id=735318405) | Acetyl-CoA carboxylase 1 | 0.03393 | -1.77 |
| [CS](http://lingenego.voeding.tno.nl:8000/cgi/gene.cgi?id=-1988765831) | Citrate synthase, mitochondrial precursor | 0.00100 | -1.34 |
| [ELOVL6](http://lingenego.voeding.tno.nl:8000/cgi/gene.cgi?id=-1748898175) | Elongation of very long chain fatty acids protein 6 | 0.01515 | -2.36 |
| [FASN](http://lingenego.voeding.tno.nl:8000/cgi/gene.cgi?id=1627788392) | Fatty acid synthase | 0.00220 | -2.02 |
| [INSIG2](http://lingenego.voeding.tno.nl:8000/cgi/gene.cgi?id=51141) | Insulin-induced gene 2 protein | 0.00054 | -1.48 |
| [IRS1](http://lingenego.voeding.tno.nl:8000/cgi/gene.cgi?id=1035396776) | Insulin receptor substrate 1 | 0.02268 | -1.37 |
| [MAPK1](http://lingenego.voeding.tno.nl:8000/cgi/gene.cgi?id=-538925915) | Mitogen-activated protein kinase 1 | 0.02675 | 1.17 |
| [PDHA1](http://lingenego.voeding.tno.nl:8000/cgi/gene.cgi?id=-1734505130) | Pyruvate dehydrogenase E1 component subunit alpha, somatic form, mitochondrial precursor | 0.01160 | -1.30 |
| [PDHB](http://lingenego.voeding.tno.nl:8000/cgi/gene.cgi?id=581204818) | Pyruvate dehydrogenase E1 component subunit beta, mitochondrial precursor | 0.01117 | -1.58 |
| [PIK3CB](http://lingenego.voeding.tno.nl:8000/cgi/gene.cgi?id=-1953999055) | Phosphatidylinositol-4,5-bisphosphate 3-kinase catalytic subunit beta isoform | 0.01382 | -1.29 |
| [PLIN](http://lingenego.voeding.tno.nl:8000/cgi/gene.cgi?id=-478881597) | Perilipin | 0.00444 | -1.75 |
| [PRKACA](http://lingenego.voeding.tno.nl:8000/cgi/gene.cgi?id=2018288499) | cAMP-dependent protein kinase, alpha-catalytic subunit | 0.02277 | -1.42 |
| [PRKAR1A](http://lingenego.voeding.tno.nl:8000/cgi/gene.cgi?id=1173076018) | cAMP-dependent protein kinase type I-alpha regulatory subunit | 0.02798 | -1.18 |
| [RAF1](http://lingenego.voeding.tno.nl:8000/cgi/gene.cgi?id=-1685026734) | RAF proto-oncogene serine/threonine-protein kinase | 0.00660 | -1.43 |
| [SCD](http://lingenego.voeding.tno.nl:8000/cgi/gene.cgi?id=41006224) | Acyl-CoA desaturase | 0.00158 | -2.05 |
| [SLC2A4](http://lingenego.voeding.tno.nl:8000/cgi/gene.cgi?id=809909675) | Solute carrier family 2, facilitated glucose transporter member 4 | 0.01228 | -2.09 |
| [SOS2](http://lingenego.voeding.tno.nl:8000/cgi/gene.cgi?id=162441899) | Son of sevenless homolog 2 | 0.01218 | -1.21 |
| [SREBF1](http://lingenego.voeding.tno.nl:8000/cgi/gene.cgi?id=763981128) | Sterol regulatory element-binding protein 1 | 0.00883 | -1.68 |
| **Glycolysis / Gluconeogenesis** | |  |  |
| Acss2 | acyl-CoA synthetase short-chain family member 2 | 0.01181 | -2.28 |
| Acyp1 | acylphosphatase 1, erythrocyte (common) type | 0.01804 | -1.22 |
| Acyp2 | acylphosphatase 2, muscle type | 0.00261 | -1.57 |
| Adh1 | alcohol dehydrogenase 1 (class I) | 0.00662 | -1.60 |
| Adh5 | alcohol dehydrogenase 5 (class III), chi polypeptide | 0.04262 | -1.19 |
| Aldh2 | aldehyde dehydrogenase 2, mitocondrial | 0.00073 | -1.44 |
| Aldoa | aldolase 1. A isoform | 0.00354 | -1.97 |
| Dlat | dihydrolipoamide S-acetyltransferase (E2 component of pyruvate dehydrogenase complex) | 0.00841 | -1.45 |
| Galm | galactose mutarotase | 0.00618 | -1.26 |
| Gpi1 | glucose phosphate isomerase 1 | 0.00332 | -1.45 |
| Hk2 | hexokinase 2 | 0.01830 | -1.69 |
| Ldha | lactate dehydrogenase A | 0.00169 | -1.81 |
| Pdha1 | pyruvate dehydrogenase E1 alpha 1 | 0.01160 | -1.38 |
| Pdhb | pyruvate dehydrogenase (lipoamide) beta | 0.01117 | -1.58 |
| Pfkp | phosphofructokinase, platelet | 0.00671 | -1.58 |
| Pgam1 | phosphoglycerate mutase 1 | 0.01385 | -1.27 |
| Pgk1 | phosphoglycerate kinase 1 | 0.01507 | -1.19 |
| Pgm2 | phosphoglucomutase 2 | 0.00372 | -1.64 |
| Tpi1 | triosephosphate isomerase 1 | 0.00668 | -1.37 |
| **Fatty acid metabolism** | |  |  |
| Acaa1a | acetyl-Coenzyme A acyltransferase 1ª | 0.02024 | -1.32 |
| Acaa1b | acetyl-Coenzyme A acyltransferase 1B | 0.04535 | -1.49 |
| Acat1 | acetyl-Coenzyme A acetyltransferase 1 | 0.01116 | -1.36 |
| Acat3 | acetyl-Coenzyme A acetyltransferase 3 | 0.03150 | -1.42 |
| Acox1 | acyl-Coenzyme A oxidase 1, palmitoyl | 0.02553 | -1.43 |
| Acox2 | acyl-Coenzyme A oxidase 2, branched chain | 0.01979 | -1.50 |
| Adh1 | alcohol dehydrogenase 1 (class I) | 0.00662 | -1.60 |
| Adh5 | alcohol dehydrogenase 5 (class III), chi polypeptide | 0.04262 | -1.19 |
| Aldh2 | aldehyde dehydrogenase 2, mitocondrial | 0.00073 | -1.44 |
| Cpt1c | carnitine palmitoyltransferase 1c | 0.02794 | 1.21 |
| Echs1 | enoyl Coenzyme A hydratase, short chain, 1, mitocondrial | 0.03414 | -1.32 |
| Hsd17b4 | hydroxysteroid (17-beta) dehydrogenase 4 | 0.00554 | -1.40 |
| **Cholesterol Biosynthesis** | |  |  |
| [ACAT1](http://lingenego.voeding.tno.nl:8000/cgi/gene.cgi?id=210749219) | Acetyl-CoA acetyltransferase, mitochondrial precursor | 0.01116 | -1.36 |
| [CYP51A1](http://lingenego.voeding.tno.nl:8000/cgi/gene.cgi?id=1389703088) | Cytochrome P450 51A1 | 0.00967 | -1.57 |
| [DHCR24](http://lingenego.voeding.tno.nl:8000/cgi/gene.cgi?id=-106366095) | 24-dehydrocholesterol reductase precursor | 0.00126 | -1.94 |
| [DHCR7](http://lingenego.voeding.tno.nl:8000/cgi/gene.cgi?id=-353534041) | 7-dehydrocholesterol reductase | 0.00603 | -1.64 |
| [FDPS](http://lingenego.voeding.tno.nl:8000/cgi/gene.cgi?id=124858506) | Farnesyl pyrophosphate synthetase | 0.01280 | -1.83 |
| [GGPS1](http://lingenego.voeding.tno.nl:8000/cgi/gene.cgi?id=2047030816) | Geranylgeranyl pyrophosphate synthetase | 0.01357 | -1.19 |
| [HMGCS1](http://lingenego.voeding.tno.nl:8000/cgi/gene.cgi?id=-1470055494) | Hydroxymethylglutaryl-CoA synthase, cytoplasmic | 0.04359 | -1.45 |
| [IDI1](http://lingenego.voeding.tno.nl:8000/cgi/gene.cgi?id=-36759211) | Isopentenyl-diphosphate Delta-isomerase 1 | 0.00431 | -1.96 |
| [LSS](http://lingenego.voeding.tno.nl:8000/cgi/gene.cgi?id=1885951515) | Lanosterol synthase | 0.00103 | -1.57 |
| [MVD](http://lingenego.voeding.tno.nl:8000/cgi/gene.cgi?id=938715670) | Diphosphomevalonate decarboxylase | 0.02238 | -1.57 |
| [MVK](http://lingenego.voeding.tno.nl:8000/cgi/gene.cgi?id=-29483516) | Mevalonate kinase | 0.02114 | -1.49 |
| [NSDHL](http://lingenego.voeding.tno.nl:8000/cgi/gene.cgi?id=1477984262) | Sterol-4-alpha-carboxylate 3-dehydrogenase, decarboxylating | 0.02461 | -1.56 |
| [PMVK](http://lingenego.voeding.tno.nl:8000/cgi/gene.cgi?id=1983200993) | Phosphomevalonate kinase | 0.00553 | -1.92 |
| [SC4MOL](http://lingenego.voeding.tno.nl:8000/cgi/gene.cgi?id=-299870649) | C-4 methylsterol oxidase | 0.00254 | -1.83 |
| [SC5DL](http://lingenego.voeding.tno.nl:8000/cgi/gene.cgi?id=204673974) | Lathosterol oxidase | 0.00095 | -2.12 |
| [SQLE](http://lingenego.voeding.tno.nl:8000/cgi/gene.cgi?id=653252974) | Squalene monooxygenase | 0.01901 | -1.69 |
| [TM7SF2](http://lingenego.voeding.tno.nl:8000/cgi/gene.cgi?id=650175602) | Delta(14)-sterol reductase | 0.00104 | -1.59 |
| **Pentose phosphate pathway** | |  |  |
| [G6PD](http://lingenego.voeding.tno.nl:8000/cgi/gene.cgi?id=-1753582738) | Glucose-6-phosphate 1-dehydrogenase | 0.00029 | -1.99 |
| [GPI](http://lingenego.voeding.tno.nl:8000/cgi/gene.cgi?id=1447992850) | Glucose-6-phosphate isomerase | 0.00531 | -1.40 |
| [H6PD](http://lingenego.voeding.tno.nl:8000/cgi/gene.cgi?id=-856851292) | GDH/6PGL endoplasmic bifunctional protein precursor [Includes: Glucose 1-dehydrogenase | 0.02796 | -1.54 |
| [PGD](http://lingenego.voeding.tno.nl:8000/cgi/gene.cgi?id=594036168) | 6-phosphogluconate dehydrogenase, decarboxylating | 0.00422 | -1.95 |
| [PGM1](http://lingenego.voeding.tno.nl:8000/cgi/gene.cgi?id=-311843672) | Phosphoglucomutase-1 | 0.00933 | -1.64 |
| [PRPS2](http://lingenego.voeding.tno.nl:8000/cgi/gene.cgi?id=-2081670195) | Ribose-phosphate pyrophosphokinase 2 | 0.02390 | 1.24 |
| [RBKS](http://lingenego.voeding.tno.nl:8000/cgi/gene.cgi?id=1337064836) | Ribokinase | 0.04642 | -1.20 |
| [RPE](http://lingenego.voeding.tno.nl:8000/cgi/gene.cgi?id=-835054681) | Ribulose-phosphate 3-epimerase | 0.00357 | -1.45 |
| [TALDO1](http://lingenego.voeding.tno.nl:8000/cgi/gene.cgi?id=-2099695797) | Transaldolase | 0.00610 | -1.80 |
| [TKT](http://lingenego.voeding.tno.nl:8000/cgi/gene.cgi?id=-92602662) | Transketolase | 0.00734 | -2.22 |
| **Glycogen pathway** | |  |  |
| [AGL](http://lingenego.voeding.tno.nl:8000/cgi/gene.cgi?id=832859156) | Glycogen debranching enzyme | 0.01184 | -1.42 |
| [GALM](http://lingenego.voeding.tno.nl:8000/cgi/gene.cgi?id=130589) | Aldose 1-epimerase | 0.00618 | -1.26 |
| [GBE1](http://lingenego.voeding.tno.nl:8000/cgi/gene.cgi?id=973167790) | 1,4-alpha-glucan-branching enzyme | 0.00565 | -1.98 |
| [GPI](http://lingenego.voeding.tno.nl:8000/cgi/gene.cgi?id=1447992850) | Glucose-6-phosphate isomerase | 0.00531 | -1.40 |
| [GYS2](http://lingenego.voeding.tno.nl:8000/cgi/gene.cgi?id=-1735578905) | Glycogen [starch] synthase, liver | 0.00488 | -1.94 |
| [HK2](http://lingenego.voeding.tno.nl:8000/cgi/gene.cgi?id=-1508624890) | Hexokinase-2 | 0.01830 | -1.69 |
| [PGM1](http://lingenego.voeding.tno.nl:8000/cgi/gene.cgi?id=-311843672) | Phosphoglucomutase-1 | 0.00372 | -1.64 |
| [PYGL](http://lingenego.voeding.tno.nl:8000/cgi/gene.cgi?id=-1332405870) | Glycogen phosphorylase, liver form | 0.00779 | -1.69 |
| [UGP2](http://lingenego.voeding.tno.nl:8000/cgi/gene.cgi?id=-1807980595) | UTP--glucose-1-phosphate uridylyltransferase | 0.00389 | -1.49 |
| **Pyruvate metabolism** | |  |  |
| Acaca | acetyl-Coenzyme A carboxylase alpha | 0.03393 | -1.77 |
| Acat1 | acetyl-Coenzyme A acetyltransferase 1 | 0.01116 | -1.36 |
| Acat3 | acetyl-Coenzyme A acetyltransferase 3 | 0.03150 | -1.42 |
| Acss2 | acyl-CoA synthetase short-chain family member 2 | 0.01181 | -2.28 |
| Acyp1 | acylphosphatase 1, erythrocyte (common) type | 0.01804 | -1.22 |
| Acyp2 | acylphosphatase 2, muscle type | 0.00261 | -1.57 |
| Akr1b8 | aldo-keto reductase family 1, member B8 | 0.02361 | -1.41 |
| Aldh2 | aldehyde dehydrogenase 2, mitocondrial | 0.00073 | -1.44 |
| Dlat | dihydrolipoamide S-acetyltransferase (E2 component of pyruvate dehydrogenase complex) | 0.00841 | -1.45 |
| Glo1 | glyoxalase 1 | 0.00795 | -1.32 |
| Hagh | hydroxyacyl glutathione hydrolase | 0.01591 | -1.47 |
| Ldha | lactate dehydrogenase A | 0.00169 | -1.81 |
| Mdh1 | malate dehydrogenase 1, NAD (soluble) | 0.00646 | -1.50 |
| Mdh2 | malate dehydrogenase 2, NAD (mitochondrial) | 0.01234 | -1.34 |
| Mod1 | malic enzyme, supernatant | 0.00280 | -2.46 |
| Pcx | pyruvate carboxylase | 0.00365 | -1.93 |
| Pdha1 | pyruvate dehydrogenase E1 alpha 1 | 0.01160 | -1.38 |
| Pdhb | pyruvate dehydrogenase (lipoamide) beta | 0.01117 | -1.58 |
| **Glycerophospholipid metabolism** | |  |  |
| Agpat3 | 1-acylglycerol-3-phosphate O-acyltransferase 3 | 0.01665 | -1.43 |
| Agps | alkylglycerone phosphate synthase | 0.02787 | -1.77 |
| Akr1c13 | aldo-keto reductase family 1, member C13 | 0.04799 | -1.20 |
| Akr1e1 | aldo-keto reductase family 1, member E1 | 0.01070 | -1.27 |
| Cds1 | CDP-diacylglycerol synthase 1 | 0.01032 | -1.42 |
| Dgke | diacylglycerol kinase, épsilon | 0.02241 | -1.18 |
| Dgkz | diacylglycerol kinase zeta | 0.04730 | 1.22 |
| Gnpat | glyceronephosphate O-acyltransferase | 0.01112 | -1.36 |
| Gpd1 | glycerol-3-phosphate dehydrogenase 1 (soluble) | 0.02890 | -1.51 |
| Hsd17b12 | hydroxysteroid (17-beta) dehydrogenase 12 | 0.01445 | -1.66 |
| Lypla1 | lysophospholipase 1 | 0.00441 | -1.28 |
| Lypla2 | lysophospholipase 2 | 0.04938 | -1.17 |
| Myst3 | MYST histone acetyltransferase (monocytic leukemia) 3 | 0.03733 | 1.24 |
| Nat6 | N-acetyltransferase 6 | 0.02152 | -1.14 |
| Pafah1b1 | platelet-activating factor acetylhydrolase, isoform 1b, beta1 subunit | 0.01836 | -1.28 |
| Pafah2 | platelet-activating factor acetylhydrolase 2 | 0.01909 | -1.27 |
| Pcyt1a | phosphate cytidylyltransferase 1, choline, alpha isoform | 0.03505 | -1.18 |
| Pcyt2 | phosphate cytidylyltransferase 2, ethanolamine | 0.02381 | -1.47 |
| Pemt | phosphatidylethanolamine N-methyltransferase | 0.00415 | -1.42 |
| Pisd | phosphatidylserine decarboxylase | 0.00152 | 1.42 |
| Pla2g12a | phospholipase A2, group XIIA | 0.04002 | -1.15 |
| Pla2g2e | phospholipase A2, group IIE | 0.00266 | -1.58 |
| Pld2 | phospholipase D2 | 0.01362 | 1.14 |
| Pnpla3 | patatin-like phospholipase domain containing 3 | 0.00098 | -2.78 |
| Rdh11 | retinol dehydrogenase 11 | 0.04622 | -1.47 |
| Rdh14 | retinol dehydrogenase 14 (all-trans and 9-cis) | 0.00378 | -1.34 |
| Sh3glb1 | SH3-domain GRB2-like B1 (endophilin) | 0.00519 | -1.42 |
