# Supplementary material for: Beta-Carotene Reduces Body Adiposity of Mice via BCMO1
Source: PLoS One. 2011 Jun 1;6(6):e20644. doi: 10.1371/journal.pone.0020644 (PMC3106009; doi:10.1371/journal.pone.0020644)
Supplement: Table S3 — Changes (p<0.05) in the expression of angiogenesis-related genes in inguinal white adipose tissue of wild-type and Bcmo1 -null mice after 14 weeks of dietary β-carotene supplementation. (DOC) [file pone.0020644.s004.doc]

**Supplementary Information**

**Beta-carotene Reduces Body Adiposity of Mice via BCMO1**

Jaume Amengual, Erwan Gouranton, Yvonne G. J. van Helden, Susanne Hessel, Joan Ribot, Evelien Kramer, Beata Kiec-Wilk, Ursula Razny, Georg Lietz, Adrian Wyss, Aldona Dembinska-Kiec, Andreu Palou, Jaap Keijer, Jean François Landrier, M. Luisa Bonet^#^ and Johannes von Lintig^#^

# Corresponding authors: M. Luisa Bonet, Laboratory of Molecular Biology, Nutrition and Biotechnology. Department of Fundamental Biology and Health Sciences, Universitat de les Illes Balears, Crta. Valldemossa Km 7.5, 07122, Palma de Mallorca, Spain. E-mail: luisabonet@uib.es and Johannes von Lintig, Department of Pharmacology, School of Medicine, Case Western Reserve University, Cleveland, OH, USA. E-mail: johannes.vonlintig@case.edu.

**Supplementary Table 3. Changes (p<0.05) in the expression of angiogenesis-related genes in inguinal white adipose tissue of wild-type and *Bcmo1*-null mice after 14 weeks of dietary β-carotene supplementation**

| **Gene** | **Gene name** | **BC diet vs control diet, wild-type mice** | **BC diet vs control diet, *Bcmo1*-null mice** |
| --- | --- | --- | --- |
|
| **Adhesion** |  |  |  |
| Ceacam1 | CEA-related cell adhesion molecule 1 | 1.60 | NC |
| Icam1 | intercellular adhesion molecule | 1.48 | NC |
| Mcam | melanoma cell adhesion molecule | -1.60 | -1.51 |
| Ncam1 | neural cell adhesion molecule 1 | 1.40 | NC |
| Jam2 | junction adhesion molecule 2 | -1.40 | NC |
| Gja5 | gap junction membrane channel protein alpha 5 | -1.18 | NC |
| Sparc | secreted acidic cysteine rich glycoprotein | -1.47 | NC |
| Cxcr4 | chemokine (C-X-C motif) receptor 4 | 1.68 | NC |
| Cx3cr1 | chemokine (C-X3-C) receptor 1 | 1.51 | NC |
| Adamts1 | a disintegrin-like and metallopeptidase (reprolysin type) with thrombospondin type 1 motif, 1 | -1.44 | NC |
| Adamts2 | a disintegrin-like and metallopeptidase (reprolysin type) with thrombospondin type 1 motif, 2 | -1.49 | NC |
| Adamts5 | a disintegrin-like and metallopeptidase (reprolysin type) with thrombospondin type 1 motif, 5 (aggrecanase-2) | -2.08 | NC |
| Adam17 | a disintegrin and metallopeptidase domain 17 | 1.48 | NC |
| Timp3 | tissue inhibitor of metalloproteinase 3 | -1.45 | NC |
| Timp4 | tissue inhibitor of metalloproteinase 4 | -2.09 | NC |
| **Growth factors** |  |  |  |
| Fgf11 | fibroblast growth factor 11 | -1.41 | NC |
| Fgf13 | fibroblast growth factor 13 | -1.95 | NC |
| Fgf21 | fibroblast growth factor 21 | -1.56 | NC |
| Fgfrl1 | fibroblast growth factor receptor-like 1 | -1.47 | NC |
| Igf1 | insulin-like growth factor 1 | NC | -1.31 |
| Hif1a | hypoxia inducible factor 1, alpha subunit | -1.40 | NC |
| Nos3 | nitric oxide synthase 3, endothelial cell | -1.50 | NC |
| Nrp1 | neuropilin 1 | NC | -1.32 |
| Vegfb | vascular endothelial growth factor B | -1.76 | NC |
| **Transcription factors** | |  |  |
| Hoxa10 | homeo box A10 | -1.46 | NC |
| Hoxa7 | homeo box A7 | -1.48 | NC |
| Hoxc6 | homeo box C6 | -1.47 | NC |
| Hoxc8 | homeo box C8 | -1.36 | NC |
| Hoxc9 | homeo box C9 | -1.56 | NC |
| **Expressed in differentiated endothelium** | |  |  |
| Ahnak | AHNAK nucleoprotein (desmoyokin) | -1.58 | NC |
| Amotl2 | angiomotin like 2 | -1.51 | NC |
| Baiap2 | brain-specific angiogenesis inhibitor 1-associated protein | -1.44 | NC |

BC, β-carotene; NC, not changed.
