# Supplementary material for: Beta-Carotene Reduces Body Adiposity of Mice via BCMO1
Source: PLoS One. 2011 Jun 1;6(6):e20644. doi: 10.1371/journal.pone.0020644 (PMC3106009; doi:10.1371/journal.pone.0020644)
Supplement: Table S4 — Beta-carotene Reduces Body Adiposity of Mice via BCMO1. (DOC) [file pone.0020644.s005.doc]

**Supplementary Information**

**Beta-carotene Reduces Body Adiposity of Mice via BCMO1**

Jaume Amengual, Erwan Gouranton, Yvonne G. J. van Helden, Susanne Hessel, Joan Ribot, Evelien Kramer, Beata Kiec-Wilk, Ursula Razny, Georg Lietz, Adrian Wyss, Aldona Dembinska-Kiec, Andreu Palou, Jaap Keijer, Jean François Landrier, M. Luisa Bonet^#^ and Johannes von Lintig^#^

**# Corresponding authors:** M. Luisa Bonet, Laboratory of Molecular Biology, Nutrition and Biotechnology. Department of Fundamental Biology and Health Sciences, Universitat de les Illes Balears, Crta. Valldemossa Km 7.5, 07122, Palma de Mallorca, Spain. E-mail: luisabonet@uib.es and Johannes von Lintig, Department of Pharmacology, School of Medicine, Case Western Reserve University, Cleveland, OH, USA. E-mail: johannes.vonlintig@case.edu.


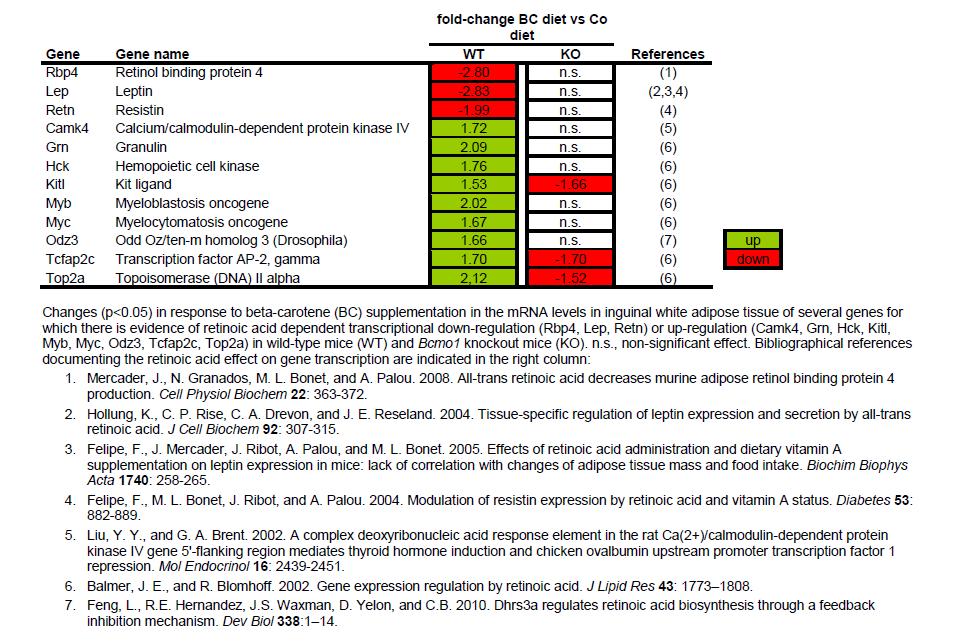
**Supplementary table 4.**
